# Host-pathogen interactions between the human innate immune system and *Candida albicans*—understanding and modeling defense and evasion strategies

**DOI:** 10.3389/fmicb.2015.00625

**Published:** 2015-06-30

**Authors:** Sybille Dühring, Sebastian Germerodt, Christine Skerka, Peter F. Zipfel, Thomas Dandekar, Stefan Schuster

**Affiliations:** ^1^Department of Bioinformatics, Friedrich-Schiller-University JenaJena, Germany; ^2^Department of Infection Biology, Leibniz Institute for Natural Product Research and Infection Biology - Hans Knöll InstituteJena, Germany; ^3^Friedrich-Schiller-University JenaJena, Germany; ^4^Department of Bioinformatics, Biozentrum, Universitaet WuerzburgWuerzburg, Germany

**Keywords:** *Candida albicans*, human immune system, host-pathogen interaction, computational systems biology, defense and evasion strategies, immunological cross-talk

## Abstract

The diploid, polymorphic yeast *Candida albicans* is one of the most important human pathogenic fungi. *C. albicans* can grow, proliferate and coexist as a commensal on or within the human host for a long time. However, alterations in the host environment can render *C. albicans* virulent. In this review, we describe the immunological cross-talk between *C. albicans* and the human innate immune system. We give an overview in form of pairs of human defense strategies including immunological mechanisms as well as general stressors such as nutrient limitation, pH, fever etc. and the corresponding fungal response and evasion mechanisms. Furthermore, Computational Systems Biology approaches to model and investigate these complex interactions are highlighted with a special focus on game-theoretical methods and agent-based models. An outlook on interesting questions to be tackled by Systems Biology regarding entangled defense and evasion mechanisms is given.

## 1. Introduction

The diploid, polymorphic yeast *Candida albicans* (Wilson et al., 2009; Kwak et al., 2014; Mech et al., 2014) is one of the most important human pathogenic fungi (Lu et al., 2014; Vylkova and Lorenz, 2014; Whittington et al., 2014). This opportunistic ubiquitous fungus (Faro-Trindade and Brown, 2009; Zipfel et al., 2011; Bain et al., 2012) usually resides as a commensal on the skin and mucosal surfaces of 30 to 70 % of the human population (Cheng et al., 2012; Jacobsen et al., 2012; Quintin et al., 2014). As part of the normal human microbiota in the gastrointestinal, oropharyngeal or urogenital tract (Moyes and Naglik, 2011; Luo et al., 2013; Wellington et al., 2014) *C. albicans* can grow, proliferate and coexist within the human host for a long time (Yan et al., 2013) without causing any symptoms of disease (Moyes and Naglik, 2011; Gow et al., 2012; Mayer et al., 2013). *C. albicans* is highly specialized for the life on or within the human host (Wilson et al., 2009). The homeostasis between *C. albicans* and the human host is kept by the human immune system (Luo et al., 2013; Yan et al., 2013; Vylkova and Lorenz, 2014) and the normal bacterial flora on mucosal surfaces and epithelial layers (Mayer et al., 2013; Yan et al., 2013; Mech et al., 2014). However, alterations in the host environment can render commensal factors into virulence attributes once the conditions favor pathogenicity (Moyes and Naglik, 2011; Bain et al., 2012; Whittington et al., 2014). Thus, there is a subtle balance between the commensal- and the pathogenic state of *C. albicans*. This is testified by a large number of defense mechanisms of the immune system and evasion mechanisms of *C. albicans*. Furthermore, there is evidence for probiotic action of *C. albicans*, for instance regarding protection of the vaginal flora (Martin et al., 1999). To understand this complex interplay, Systems Biology approaches have shown to be very instrumental (Hummert et al., 2010; Mech et al., 2014; Tierney et al., 2014). Here we provide a systematic overview of the host-pathogen interactions to promote this endeavor.

There are two major types of *C. albicans* infections in humans (Filler, 2013; Luo et al., 2013; Mayer et al., 2013). Superficial mucosal diseases like vaginal or oral candidiasis are extremely common (Cheng et al., 2012; Filler, 2013; Wellington et al., 2014). It is estimated that 75 % of all women worldwide suffer from vulvovaginal candidiasis at least once in their life and 40 to 50 % experience recurrent infections (Wilson et al., 2009; Mayer et al., 2013). Startlingly vaginal infections often occur without any sign of immune defect (Jacobsen et al., 2012). While *Candida*-associated denture stomatitis is caused in elderly and edentulous individuals (Moyes and Naglik, 2011; Mayer et al., 2013) oral and oesophageal candidiasis is particularly common in HIV-positive individuals (Wilson et al., 2009; Moyes and Naglik, 2011; Jacobsen et al., 2012). Severe mucosal diseases and life-threatening systemic infections arise in immunocompromised individuals (Yan et al., 2013; Mech et al., 2014; Wellington et al., 2014). These invasive infections of the bloodstream and virtually every organ of the human body (Wilson et al., 2009; Mayer et al., 2013; Vylkova and Lorenz, 2014) are associated with a severe morbidity (Faro-Trindade and Brown, 2009; Zipfel et al., 2011; Cheng et al., 2012; Luo et al., 2013), an unexeptably high mortality (Zipfel et al., 2011; Filler, 2013; Yan et al., 2013) and high healthcare costs (Yan et al., 2013; Vialas et al., 2014). As disseminated hematogenous candidiasis is the 3rd to 4th most common nosocomial bloodstream infection (Faro-Trindade and Brown, 2009; Wilson et al., 2009; Vylkova and Lorenz, 2014) *C. albicans* is medically as important as many mainstream bacterial infections including *Enterococci* like *Escherichia coli* and *Pseudomonas* spp. (Moyes and Naglik, 2011; Zipfel et al., 2011).

It is of particular significance that the human immune system is able to discriminate between the commensal colonization and the pathogenic invasion phase of *C. albicans* (Cheng et al., 2012; Gow et al., 2012). A robust immune response is therefore required to protect the host against *Candida* infection (d'Enfert, 2009; Jacobsen et al., 2012). This immune response can be divided into physical barriers and immune-barriers of the mucosa (Luo et al., 2013; Yan et al., 2013). The complexity of possible host-pathogen-interactions is high, as *C. albicans* is likely to encounter different components of the human immune system (Jacobsen et al., 2012). *C. albicans* has developed a large number of strategies to evade or undermine the antimicrobial defense responses of the host immune system (Collette and Lorenz, 2011; Zipfel et al., 2011; Lopez, 2013; Luo et al., 2013). These strategies may allow the fungus to control the host immune attack, to cross tissue barriers and to disseminate in the human body (Zipfel et al., 2011; Jacobsen et al., 2012; Luo et al., 2013).

In this review, we describe the immunological cross-talk between *C. albicans* and the human immune system. We follow the trail of infection: starting from phenotypic adaptations and morphogenesis, *Candida* encounters stress by the host and its infected tissue environment including nutrient limitation, temperature and pH stress. Furthermore, the host immune system responds with the innate immune attack as the immediately acting primary line of defense against systemic fungal infections (Cheng et al., 2012; Lopez, 2013). That line of host defense mainly relies on humoral complement actions, antimicrobial peptides and the cellular response mediated by phagocytes, especially by neutrophils and macrophages (Bain et al., 2012; Cheng et al., 2012; Jiménez-López and Lorenz, 2013).

Because of the complex immune response we here focus on the innate immune system. The adaptive immune system contributes with many additional mechanisms (Curtis and Way, 2009; Korn et al., 2009; Hamad, 2012) which are beyond the scope of this review. For a review on the crosstalk between innate and adaptive immune-response and the role of dendritic cells see Hamad (2012). Here, we start to give an overview of the crosstalk of human defense mechanisms and the corresponding fungal evasion mechanisms. As the total amount of such mechanisms is enormous, the list cannot be exhaustive.

A number of different Systems Biology approaches exist to model and simulate host pathogen interactions, e.g., Boolean modeling (Naseem et al., 2012; Schlatter et al., 2012) and reverse engineering (Tierney et al., 2012). However, no matter which strategy is chosen, there is always a game of life and death involved and hence game theoretical approaches and agent-based modeling are particularly powerful and thus reviewed here. These approaches are useful to depict the highly complex and dynamic host-pathogen interactions and can help to gain further insights into the underlying processes of *C. albicans* infections.

## 2. Host defense and corresponding fungal evasion strategies

As a commensal as well as an invading pathogen *C. albicans* faces stressors of the host environment. Those stress sources include changes in nutrient availability, pH, osmolarity, temperature, or attack by the cells of the immune system (Wilson et al., 2009). *C. albicans* has a robust stress response mediated by humoral components as well as rapid alterations in gene expression of stress-responsive regulatory pathways which allow *C. albicans* to respond to changes of environmental stimuli (Wilson et al., 2009; Mayer et al., 2013).

### 2.1. Stress induced by the human host and its environment

As a commensal *C. albicans* competes with all the probiotic microorganisms of the host's microflora for nutrients (Brunke and Hube, 2013; Whittington et al., 2014). Even though the gut is relatively rich in nutrients, those nutrients are quickly absorbed by the microbial flora and the epithelial cells (Whittington et al., 2014). In other host niches nutrients are limited by the host and usually not available to pathogens (see Table 1). However, *C. albicans* is metabolically flexible and uses nutrient acquisition mechanisms such as sequestration of iron and zinc to survive and grow in the many different and changing host niches such as the gastrointestinal, oropharyngeal or urogenital tract (Brunke and Hube, 2013; Whittington et al., 2014).

**Table 1:** **Pairs of defense and evasion strategies—adapting to the host environment**.

**Human host**	***C. albicans***
Limiting nutrient availability to pathogens	Release of secreted aspartic proteases (Saps) to liberate oligopeptides and amino acids from tissues
Nutrient starvation e.g., in phagocytes	Switching from the glycolytic pathway to the glyoxylate cycle and gluconeogenesis to metabolize alternative carbon sources
Active sequestration of iron	Iron acquisition through: a reductive system, a siderophore uptake system and a heme-iron uptake system
Active sequestration of zinc	Zinc acquisition via a zincophore system
Inducing pH-stress	Sense and adapt to environmental pH (Pra1); modulate extracellular pH by actively alkalizing the surrounding environment
Inducing thermal stress like fever	Heat shock response mediated by heat shock proteins and trehalose accumulation
Inducing osmotic stress	Outer cell wall structure as protection from osmotic pressure; intracellular accumulation of glycerol to counteract the loss of water

One of those mechanisms is the release of secreted aspartic proteases (Saps) by *C. albicans*. Saps can destroy host tissue and liberate oligopeptides and amino acids. These liberated carbon sources are then taken up by *C. albicans* via oligopeptide and amino acid transporters (Brunke and Hube, 2013). Adding to the metabolic flexibility *C. albicans* has no known auxotrophies and can metabolize a broad range of sugars and all amino acids (Brunke and Hube, 2013; Lopez, 2013).

When facing nutrient starvation or phagocytosis *C. albicans* shows responses that are similar in the two cases, switching from the glycolytic pathway to the glyoxylate cycle and gluconeogenesis (Faro-Trindade and Brown, 2009; Lopez, 2013; Vylkova and Lorenz, 2014) which is absent from humans. This metabolic shift enables *C. albicans* to metabolize alternative, less favored carbon sources (De Figueiredo et al., 2008; Faro-Trindade and Brown, 2009). Next to amino acids and lipids, lactate produced by tissues and bacteria in the gut serves as one potential carbon source (Lopez, 2013). The growth on alternative carbon sources can cause substantial changes in the cell wall of *C. albicans* even when the morphology of the cell is otherwise unaltered (Gow et al., 2012). This influences the recognition by phagocytes as well as drug- and stress-resistance of the cell (Vylkova and Lorenz, 2014). *C. albicans* cells grown on lactate have been shown to be more resistant to osmotic, envelope and antifungal stresses and to be more adherent. They even elicit lower levels of proinflammatory cytokines from monocytic cells and once phagocytosed are more harmful to macrophages than *C. albicans* cells grown on glucose. The exposure to non-preferred carbon sources therefore benefits *C. albicans*, especially in their interactions with macrophages (Lopez, 2013). This fact may be helpful for metabolic therapy strategies, as supplementing certain nutrients in the infection locus may render *C. albicans* more vulnerable to the host defense.

Another important defense mechanism in the host's “nutritional immunity” is the active sequestration of metals (Brunke and Hube, 2013). The most important micro nutrients that are prerequisite for *C. albicans* infection are iron, zinc, manganese and copper (Brunke and Hube, 2013; Mayer et al., 2013). Both the pathogenic fungus and its host have evolved mechanisms to acquire and restrict access to these metals (Mayer et al., 2013).

The human host is severely restricting the availability of iron to pathogens by keeping the iron levels of the blood and the tissue environment low (Brunke and Hube, 2013). This is achieved by storing iron in iron-binding proteins like ferritin, lactoferrin, hemoglobin and transferrin which are usually not accessible to pathogenic microbes (Faro-Trindade and Brown, 2009; Jacobsen et al., 2012). *C. albicans* on the other side has developed a plethora of iron acquisition systems (Brunke and Hube, 2013) including a reductive system, a siderophore uptake system and a heme-iron uptake system (Mayer et al., 2013). *C. albicans* can utilize its siderophore uptake system via Sit1/Arn1 (siderophore iron transport 1) to steal iron from siderophores produced by other microorganisms without producing its own siderophores (Brunke and Hube, 2013; Mayer et al., 2013). *C. albicans* can further bind host ferritin with the hyphae-associated adhesion and invasion protein Als3 (agglutinin-like sequence 3) (Jacobsen et al., 2012; Brunke and Hube, 2013; Mayer et al., 2013). The reductive system, with its large gene families of reductases, oxidases and iron permeases (Brunke and Hube, 2013), then mediates the iron acquisition from host ferritin, transferrin or if available free iron from the environment. *C. albicans* can also use iron from host hemoglobin and hemoproteins by first expressing haemolysins that disrupt red blood cells (Brunke and Hube, 2013; Mayer et al., 2013). Subsequently the iron acquisition is mediated by the heme-receptor gene family members RBT5, RBT51, CSA1, CSA2, and PGA7 (RBT6) (Mayer et al., 2013).

Zinc as a central cofactor for many proteins is an abundant metal in most living organisms (Brunke and Hube, 2013). The sequestration of zinc is therefore a potent antifungal mechanism of the host during infections and is mediated by calprotectin (Faro-Trindade and Brown, 2009; Brunke and Hube, 2013). *C. albicans* can acquire zinc via a “zincophore” system using pH-regulated antigen 1 (Pra1) (Brunke and Hube, 2013). Secreted Pra1 acts as a zincophor, similar to iron-carrying siderophores (Brunke and Hube, 2013), binds extracellular zinc and reassociates with the fungal cell (Mayer et al., 2013). This reassociation is mediated by the zinc transporter Zrt1 (Mayer et al., 2013).

Though copper and manganese are essential for fungal growth, the mechanisms by which *C. albicans* acquires them are less well understood. There is a putative manganese transporter, Ccc1, and a putative copper transporter, Ctr1, but their roles in *C. albicans* virulence have not yet been determined (Mayer et al., 2013).

Next to nutritional stress, pH-stress is of fundamental importance to *C. albicans*. Depending on the host niche *C. albicans* encounters many different pH levels. While the pH of human blood and tissues is slightly alkaline, the pH of the digestive tract ranges from very acidic to more alkaline. pH-stress can also occur in the urogenital tract as well as in the phagolysosome, where pH is very acidic, once *C. albicans* is phagocytosed by cells of the innate immune system. However, *C. albicans* is able to adapt to significant changes in its surrounding pH. The two *C. albicans* cell wall proteins Phr1 (pH responsive 1) (required for systemic infections) and Phr2 (essential for infections of the vagina) are important for adaptation to changing pH. Astonishingly *C. albicans* is not only able to sense and adapt to environmental pH but also to modulate extracellular pH by actively alkalizing its surrounding environment (Mayer et al., 2013). *C. albicans* can release ammonia derived from amino acid degradation to raise extracellular pH (Lopez, 2013). This is of special importance after phagocytosis as it promotes the neutralization of the phagosomal pH, inducing hyphal morphogenesis and thereby fosters the escape of the pathogen from macrophages (Vylkova and Lorenz, 2014).

Thermal stress like fever and cold leads to a heat shock response mediated by heat shock proteins and trehalose accumulation in *C. albicans*. These heat shock proteins and trehalose act as “molecular- and chemical- chaperons” by preventing deleterious protein unfolding and aggregation (Mayer et al., 2013).

The outer layer of *C. albicans'* cell wall not only defines the cell shape and provides an efficient barrier against immune reactions but also protects the fungus from osmotic pressure (Luo et al., 2013). A further osmotic stress response is the intracellular accumulation of glycerol to counteract the loss of water due to an outward-directed chemical gradient. The glycerol biosynthesis is mediated by the glycerol 3-phosphatase (Gpp1) and the glycerol 3-phosphate dehydrogenase 2 (Gpd2) (Mayer et al., 2013).

### 2.2. The human innate immun system

Zipfel et al. (2011) started a list of immune evasion and tissue invasion mechanisms including complement evasion, evasion of cellular response and tissue invasion mechanisms by *C. albicans* which we include and further augment in Table 2. Several of the mechanisms listed in Table 2 have been modeled by Systems Biology approaches (see Section 3).

**Table 2:** **Pairs of defense and evasion strategies—*C. albicans* and the human innate immune system**.

**Human host**	***C. albicans***
**EPITHELIAL RESPONSE**	
Physical barrier	Active penetration by thigmotropism, elongating hyphae and production of lytic enzymes; induction of endocytosis; degradation of extracellular matrix component by recruiting human plasminogen to the yeast surface and secretion of lytic enzymes
Chemical barrier in form of secreted antimicrobial peptides and degradative enzymes	Respond to β-defensin activity via the high-osmolarity glycerol (HOG) pathway; secretion of Sap9 and a Msb2 fragment
The host uses *C. albicans'* pumps to get antimicrobial peptides into the pathogen	Uses multi-drug resistance pumps such as Flu1 to transport antimicrobial peptides out of the pathogen
**COMPLEMENT RESPONSE**	
Complement systems barrier	Acquiring human complement regulators to the cell surface; secretion of complement inhibitors to block C3 complement activation; production of proteases (Saps) to degrade host complement proteins
**CELLULAR RESPONSE**	
PRRs recognition barrier via dectin-1, dectin-2, etc.	Surface mannans shield β-glucan from recognition by dectin-1 to avoiding phagocytosis; release of soluble decoys to evade host immune responses
Barrier in form of pro- and anti-inflammatory cytokines and chemokines production	Inhibition of proinflammatory IL-17 production by altering the host tryptophan metabolism; induction of anti-inflammatory cytokine release
Inhibition of *C. albicans* yeast-to-hyphal transition by neutrophils	No known evasion mechanism
Cellular ET formation by neutrophils and macrophages	No known evasion mechanism
Phagocytosis	Biofilm formation; inhibition of phagolysosome formation; neutralization of phagosomal pH inside macrophages; induction of hyphal morphogenesis and escape from the immune cell in macrophages and natural killer cells; pyroptosis / macrophage cell death
Oxidative and nitrosative stress induced by neutrophils and macrophages	Inhibition of ROS generation by macrophages through an unknown mechanism; secretion of Sod enzymes, catalases, glutathione peroxidases and thioredoxin to detoxify extracellular ROS; accumulation of trehalose against oxidative stress; production of intracellular flavohemoglobin enzymes against nitrosative stress; biofilm formation

*C. albicans* can switch readily between yeast, hyphal and pseudohyphal growth and back (Brunke and Hube, 2013; Kwak et al., 2014; Lu et al., 2014). Both the yeast and hyphal forms of the fungus are required for biofilm formation as well as full virulence (Lopez, 2013; Yan et al., 2013). Mutants locked in one morphology are avirulent and show a significantly reduced growth performance in biofilm formation (Baillie and Douglas, 1999; Lopez, 2013; Yan et al., 2013).

Biofilms are three-dimensional microbial communities in an extracellular matrix adhering to mucosal or artificial surfaces (Ganguly and Mitchell, 2011) for example biomaterials used for implants like stents and catheters. While the *C. albicans* biofilms on abiotic surfaces consist of yeast and filamentous cells of the fungus (Baillie and Douglas, 1999; Ramage et al., 2006; Ganguly and Mitchell, 2011) the *in vivo C. albicans* biofilms are polymicrobial with an extracellular matrix layer that contains host immune cells (Ganguly and Mitchell, 2011). *C. albicans* biofilms protect the pathogen from host immune attacks and antifungal drugs (Baillie and Douglas, 1998; Seneviratne et al., 2008; Yan et al., 2013). Especially *C. albicans* abiotic surface biofilms are associated with increased drug resistance (Baillie and Douglas, 1998; Ganguly and Mitchell, 2011). This antifungal resistance increases with biofilm maturation (Chandra et al., 2001). There are indications that *C. albicans* biofilms are even resistant to killing by neutrophils (Ganguly and Mitchell, 2011; Mayer et al., 2013) and do not trigger the production of reactive oxygen species (ROS) (Mayer et al., 2013). Reviews on the regulatory control of *C. albicans* within biofilms can be found in Nobile and Mitchell (2006) and Finkel and Mitchell (2011). For a review on *C. albicans* biofilms on mucosal surfaces see Ganguly and Mitchell (2011).

After invading host tissues *C. albicans* encounters an early defense line: the innate immune system. The innate immune system maintains host homeostasis by recognizing and cleaning modified or damaged host cells. It directly attacks and limits the growth of invading microbes without inflammatory reactions. This defense is mediated through three major effector mechanisms: antimicrobial peptides, the complement system and immune cells that recognize and respond to foreign microbes (Zipfel et al., 2011).

The initial interaction of *C. albicans* with the human immune system is with epithelial cells of the mucosa (Moyes and Naglik, 2011; Luo et al., 2013) that act as physical barriers. The fungus is able to invade the human host tissues via two routes: induced endocytosis and active penetration (Mech et al., 2014). The passive uptake is a host driven process, mediated by *C. albicans* surface proteins Als3 and Hgc1 (hypha-specific expression and relatedness to G1 cyclins 1) which bind to epithelial cell E-cadherin (Wilson et al., 2009). Active penetration on the other hand does not rely on the host but exclusively on fungal attributes including physical pressure applied by the advancing hyphal tip, thigmotropism and the secretion of extracellular hydrolases like Saps, class B phospholipase (Plb) and lipase (Lip) families (Wilson et al., 2009; Mayer et al., 2013).

Epithelial cells not only provide a physical barrier but also have an active, integral role in mucosal protection against *C. albicans* by discriminating between the commensal and pathogenic form of the fungus. Next to the NF-κB pathway, Moyes and Naglik (2011) identified the MAPK signaling as an important mechanism in the epithelial cell responses to *Candida* infections. The presence of *C. albicans* yeast or hyphae triggers the NF-κB signaling and an early response of the MAPK activation through ERK1/2 and JNK signaling which induces the c-Jun activity. When a sufficient fungal hyphal burden is present and the threshold level of activation is reached, a second prolonged, late response is induced, activating MAPK regulation via the MAPK phosphatase MKP1 through ERK1/2 and p38 signaling. This in turn induces c-Fos activity resulting in the production of cytokines with a proinflammatory profile like interleukin 1α/β(IL-1α/β), IL-6, G-CSF, GM-CSF, and TNF-α as well as the chemokines RANTES, IL-8, and CCL20 (Steele and Fidel, 2002; Moyes and Naglik, 2011; Cheng et al., 2012). Chin et al. (2014) showed that the post-infection regulation of cytokines for IL-2, IL-6, TNF-α, TNF-β are organ-specific (i.e., kidney, spleen, brain). The secretion of proinflammatory molecules results in the recruitment, differentiation, and activation of various immune cells (Moyes and Naglik, 2011; Cheng et al., 2012). Especially important for an early immune response of mucosal surfaces to *C. albicans* infection is IL-22. This cytokine is produced by innate and adaptive immune cells (De Luca et al., 2010; Zenewicz and Flavell, 2011). A heterodimeric receptor consisting of IL-22R and IL-10Rb recognizes IL-22 (Eyerich et al., 2011; Sonnenberg et al., 2011; Zenewicz and Flavell, 2011). As the expression of IL-22R is mainly confined to epithelial cells, the signaling is specific to tissues (Eyerich et al., 2011; Zenewicz and Flavell, 2011). IL-22 has both pro- and anti-inflammatory functions (De Luca et al., 2010) and stimulates the proliferation (Kagami et al., 2010; Zenewicz and Flavell, 2011) and together with IL-17 the production of antimicrobial peptides by epithelial cells (De Luca et al., 2010; Kagami et al., 2010; Eyerich et al., 2011). The IL-23/IL-22 axis controls the initial fungal growth and tissue homeostasis (Luca, Zelante, D'angelo, Zagarella, Fallarino and Spreca, 2010; Luca and Romani, 2011). The combinatorial secretion of IL-22 and TNF-α by Th22 cells increases the induction and secretion of the complement factors C1r and C1s, antimicrobial chemokines and antimicrobial peptides (Eyerich et al., 2011).

#### 2.2.1. Antimicrobial peptides

Two important groups of antimicrobial peptides are α- and β-defensins. The α-defensins group consists of four cationic peptides, HNP1 to HNP4, that are found in the azurophilic granules of human neutrophils. The group of β-defensins is primarily expressed by epithelial cells and includes human β-defensins 2 and 3 (hBD-2 and hBD-3), that have significant antifungal activity. They can be induced by a variety of agents, including TLR agonists, as well as monocyte- and macrophage-derived factors, such as IL-1 (Faro-Trindade and Brown, 2009). The two groups of α- and β-defensins can be distinguished based on their arrangement of disulfide linkages (Faro-Trindade and Brown, 2009; Yan et al., 2013). Both defensin groups target *C. albicans* cell membranes and cause nonlytic permeabilization and release of cellular ATP (Faro-Trindade and Brown, 2009). The damage imposed on *C. albicans* by hBD-2 and hBD-3 shares similarities with that caused by osmotic and oxidative stress. *C. albicans* in turn can respond to these hBD-2 and hBD-3 injuries via the high-osmolarity glycerol (HOG) pathway and rescue cells from β-defensin activity (Yan et al., 2013). Defensins can also act as chemoattractants for monocytes, dendritic cells, and selected lymphocytes (Faro-Trindade and Brown, 2009).

Another important antimicrobial peptide is LL-37, that kills *C. albicans* by fragmenting the cellular membrane of the fungus, leading to efflux of molecules like ATP and proteins (Den Hertog et al., 2005; Faro-Trindade and Brown, 2009). LL-37 can further act as a chemoattractant for neutrophils, monocytes and lymphocytes, induce histamine release from mast cells, alter the transcriptional response in macrophages and play a role in wound repair (Faro-Trindade and Brown, 2009). The peptide is produced by the proteolytic cleavage of cathelicidin (hCAP-18) (Den Hertog et al., 2005; Faro-Trindade and Brown, 2009). The hCAP-18 produced in neutrophils, and other cells including monocytes, natural killer cells, lymphocytes and a variety of epithelial cells, has antimicrobial activity itself (Faro-Trindade and Brown, 2009).

A family of cationic serine proteases called serprocidins also possess antimicrobial activity. Members of this family are stored within neutrophil granules and include protease-3, cathepsin G, and elastase. Those proteins are involved in many cellular processes including the cleavage of hCAP-18, cellular activation, as well as chemotaxis (Faro-Trindade and Brown, 2009).

Another example of an antimicrobial enzyme is lysozyme which targets the cell membrane of *C. albicans*. Lysozyme is expressed by a variety of phagocytes, including granulocytes, monocytes as well as macrophages and can be found at high levels in various tissues and secretions such as saliva. Its fungicidal activity is thought to occur through enzymatic hydrolysis of N-glycosidic bonds within the fungal cell wall and injury to the cell membrane (Faro-Trindade and Brown, 2009).

It is worth noting that the host makes use of *C. albicans'* polyamine influx transporters to get some antimicrobial peptides like histatin 5 into the pathogen. *C. albicans* in turn, uses multi-drug resistance pumps such as the fungal polyamine efflux transporter Flu1 to transport those antimicrobial peptides out again and thus reduce their toxicity (Li et al., 2013). *C. albicans* is also able to cleave histatin 5 with its protease Sap9 (Szafranski-Schneider et al., 2012).

Another mechanism by which *C. albicans* deals with antimicrobial peptides is the shedding of a large glycosylated fragment of Msb2. *C. albicans'* Msb2 stabilizes the fungal cell wall and inactivates histatin 5 and LL-37 (Szafranski-Schneider et al., 2012; Swidergall et al., 2013) as well as human α- and β-defensins (Swidergall et al., 2013).

#### 2.2.2. Complement system

The complement system is highly efficient in recognizing and eliminating infectious pathogens while its activation is tightly regulated in time and space. For reviews about the interactions of *C. albicans* with the human complement system see Zipfel et al. (2011); Cheng et al. (2012); Zipfel et al. (2013); Luo et al. (2013).

For complement evasion *C. albicans* acquires several human complement regulators, e.g., C4BP (complement component 4b-binding protein), factor H, FHL-1 (four and a half LIM domains protein 1), plasminogen and vitronectin, to its cell surface to inhibit the actions of the complement system (Luo et al., 2013). Factor H is bound by four *C. albicans* proteins: phosphoglycerate mutase (Gpm1), Pra1, the high-affinity glucose transporter 1 (Hgt1p) and Gpd2 (Luo et al., 2013; Zipfel et al., 2013). *C. albicans* Pra1 and Hgt1p also bind C4BP. There are eleven *C. albicans* proteins that bind host plasminogen: Gpm1, enolase, Tsa1, Cta1 (catalase 1), Tdh3 (triose phosphate dehydrogenase 3), Tef1 (translation elongation factor 1-alpha), Pgk1 (phosphoglycerate kinase 1), Adh1 (alcohol dehydrogenase 1), Fba1 (fructose-bisphosphate aldolase), Pra1 and Gpd2 (Zipfel et al., 2013) and three *C. albicans* proteins which bind human FHL-1: Gpm1, Pra1 and Gpd2 (Luo et al., 2013). Human plasminogen bound on *C. albicans'* cells, can be activated to proteolytically active plasmin that cleaves host fibrinogen thereby contributing to the tissue invasion of *C. albicans* cells into epithelia cell layers (Zipfel et al., 2011). *C. albicans* further expresses ανβ3 integrin-like protein that acquires host vitronectin to the fungal cell surface. This in turn inhibits the formation of the terminal complement complex (Luo et al., 2013). *C. albicans* can furthermore secrete aspartyl proteases Sap1, Sap2, and Sap3 that degrade the host complement proteins C3b, C4b and C5 (Gropp et al., 2009; Luo et al., 2013). The expression of endogenous complement inhibitors like secreted Pra1 which binds the central complement component C3 in solution is another mechanism by *C. albicans* to block C3 and complement activation (Zipfel et al., 2011). Secreted Pra1 also blocks the human integrin receptors CR3 and CR4, expressed by human leukocytes, granulocytes, macrophage and natural killer cells thereby inhibiting recognition, phagocytosis and cell-mediated killing (Luo et al., 2013).

Phagocytes respond to pathogens by recognizing opsonins and pathogen-associated molecular pattern (PAMPs) using surface expressed pattern recognition receptors (PRRs) (Jacobsen et al., 2012; Lopez, 2013; Luo et al., 2013). As the cell wall of *C. albicans* contains carbohydrates and cell wall proteins that are not present in the human body, it represents an ideal immunological target (Gow et al., 2012). Exhaustive reviews on *C. albicans'* cell wall architecture and its recognition have been published by Netea et al. (2008); Netea and Maródi (2010); Moyes and Naglik (2011); Gow et al. (2012). The most important *C. albicans* PAMPs are its cell wall carbohydrates: mannan (as mannosylated proteins), β-glucan, and chitin (Lopez, 2013). One mechanism used by *C. albicans* to evade the cellular response by phagocytes is to shield these β-glucans with surface mannans upon hyphal growth to avoid phagocytosis (Luo et al., 2013). The receptor ligation of PRRs with PAMPs activates resident phagocytes and leads to synthesis and secretion of cytokines and lipid mediators. One evasion mechanism by *C. albicans* is the induction of an anti-inflammatory cytokine release by favoring toll-like receptor (TLR) 2 instead of TLR4 recognition (Zipfel et al., 2011). *C. albicans* is further able to inhibit the proinflammatory IL-17 production by altering the host tryptophan metabolism. This metabolism is regulated by two distinct enzymes: Indoleamine 2,3- dioxygenase (IDO) and tryptophan hydroxylase. By inhibiting IDO expression, *C. albicans* can shift the tryptophan metabolism, leading to fewer kynurenines and more 5-hydroxytryptophan metabolites. The increased 5-hydroxytryptophan levels subsequently inhibit the host IL-17 production (Cheng et al., 2010, 2012). A similar mechanism is used by cancer cells (Uyttenhove et al., 2003) and has been described by a mathematical model (Stavrum et al., 2013). For a detailed explanation of the recognition of *C. albicans* PAMPs and the *C. albicans* evasion strategies from epithelial cell defense see Netea et al. (2008); Netea and Maródi (2010); Moyes and Naglik (2011); Gow et al. (2012); Mech et al. (2014).

#### 2.2.3. Phagocytes

While viral infections are primarily fought by T-cells in particular T-killer-cells, defense against fungi resembles bacteria defense in mobilizing neutrophils and macrophages.

Invading and disseminating *C. albicans* cells are faced with phagocytic cells (Kumar and Sharma, 2010; Zipfel et al., 2011; Jacobsen et al., 2012). Phagocytes, especially neutrophils and macrophages are of major importance for the host defense against mucosal and disseminated candidiasis (Cheng et al., 2012; Krysan et al., 2014; Quintin et al., 2014). These immune cells most effectively control and clear *C. albicans* infections by killing *C. albicans* cells intracellularly and extracellularly (Cheng et al., 2012). *C. albicans* on the other hand has evolved several mechanisms to control and evade the antimicrobial activity of local and newly attracted phagocytic cells by inhibiting recognition, trafficking, and effector release, thus overcoming several important stresses (Lopez, 2013; Luo et al., 2013).

Neutrophils are the prevalent immune cell type in anti-*Candida* immunity (Moyes and Naglik, 2011; Luo et al., 2013). During *C. albicans* infection, neutrophils migrate to sites of infection and release one or more chemotactic factors (Luo et al., 2013). After recognition of *C. albicans* cells through dectin-1 (recognizes β-1,3 glucan), dectin-2 (recognizes mannan), TLR2 (recognizes phospholipomannan), TLR4 (recognizes O-mannan), and mannose receptor (recognizes N-mannan) (Moyes and Naglik, 2011) neutrophils induce epithelial cell mediated protection against *C. albicans* infections and can directly kill *Candida* cells (Moyes and Naglik, 2011). The presence of neutrophils further inhibits *C. albicans* growth, including the yeast-to-hyphal transition (Jacobsen et al., 2012). These immune cells preferentially target *C. albicans* hyphae but kill yeast and hyphal forms of *C. albicans* at the same rate (Jacobsen et al., 2012; Tyc et al., 2014). Neutrophils rely on a range of antimicrobial effector mechanisms including oxidative burst, cytokine release, phagocytosis, neutrophil extracellular traps (NETs), release of granule enzymes as well as antimicrobial peptides to kill the fungus (Luo et al., 2013). Additionally they may differentiate into discrete subsets defined by distinct phenotypic and functional profiles (Scapini and Cassatella, 2014).

Another important immune cell type in anti-*Candida* immunity are macrophages (Jiménez-López and Lorenz, 2013; Krysan et al., 2014; Liu et al., 2014). These dynamic cells are distributed in various tissues and are part of the first line of host defense (Brunke and Hube, 2013; De Lima et al., 2014; Liu et al., 2014). Macrophages are of particular importance as they can both limit *C. albicans* burden early in infection and recruit and activate other immune effector cells (Krysan et al., 2014). Macrophages produce a variety of pro- and anti-inflammatory cytokines and chemokines in response to *C. albicans* (Jacobsen et al., 2012; Krysan et al., 2014). Particularly, *C. albicans* hyphae formation is a strong trigger for the production of IL-1β (Krysan et al., 2014) thereby helping to orchestrate the immune responses of the host (Jacobsen et al., 2012; Brunke and Hube, 2013). Cheng et al. (2011) showed that the development of hyphae during tissue invasion triggers the recognition by macrophages via the dectin-1/inflammasome pathway, leading to IL-1β production and thus T helper cell 17 (Th17 cell) activation. For a review on inflammasome activation see van de Veerdonk et al. (2015). Macrophages damage or directly kill *C. albicans* (Krysan et al., 2014) utilizing a combination of oxidative and nonoxidative microbicidal mechanisms including the production of antimicrobial peptides and degradative enzymes, the generation of ROS and nitric oxide synthase (iNOS), phagocytosis and macrophage extracellular traps (METs) (Liu et al., 2014). During phagocytosis macrophages readily ingest the round yeast form of *C. albicans* as well as relatively short filaments (Jacobsen et al., 2012; Brunke and Hube, 2013; Krysan et al., 2014). The fungus on the other side has developed several defense strategies to escape from macrophages with a significant cytotoxic effect on the immune cell, e.g., pyroptosis (Krysan et al., 2014).

Natural killer cells are innate lymphocytes with a potent cytotoxic activity. They usually are of major importance in viral infections and anti-tumor immunity (Voigt, 2013). The role of natural killer cells in host defense against *C. albicans* infection strongly differs depending on the state of host defense. While natural killer cells are an essential and non-redundant component of anti-*C. albicans* host defense in immunosuppressed hosts with defective T- and B-lymphocyte immunity they can contribute to hyperinflammation in immunocompetent hosts (Quintin et al., 2014). Natural killer cells modulate the immune responses by secreting cytokines which in turn recruit and activate other innate immune cells. Natural killer cells are also able to phagocytose *C. albicans* cells. However, in contrast to the professional phagocytic activity of neutrophils, this does not inhibit the further elongation of *C. albicans* filaments and leads to the destruction of the natural killer cell (Voigt, 2013). It was therefore proposed by Voigt (2013) that these immune cells contribute to the protective immunity against *C. albicans* by recruiting other immune cells and enhancing proinflammatory activities without efficiently restricting the fungus.

Another important innate immune cell type for the *C. albicans* defense are dendritic cells. They are professional antigen-presenting cells which coordinate the immune response and link innate and adaptive immunity (Cheng et al., 2012; Ramirez-Ortiz and Means, 2012). Dendritic cells reside and patrol in the skin and mucosal surface and ingest *Candida* once tissues are invaded (d'Ostiani et al., 2000; Cheng et al., 2012). These immune cells use C-type lectin pattern recognition receptors like Dectin-1, Dectin-2 and DC-Sign to recognize the fungus. Dendritic cells phagocytose both yeast and hyphal *C. albicans* cells but kill yeast cells more efficiently (Jacobsen et al., 2012). After processing *C. albicans* they present *Candida*-specific antigens via major histocompatibility complex class II molecules (Cheng et al., 2012). Dendritic cells therefore have a bridging effect between the innate and adaptive antifungal responses. They are able to discriminate between yeast- and hyphal- forms of *C. albicans* (d'Ostiani et al., 2000; Cheng et al., 2012) and induce different T helper cell differentiation depending on the morphology of phagocytosed *C. albicans* cells (d'Ostiani et al., 2000; Cheng et al., 2012; Jacobsen et al., 2012). While yeast cells stimulate the priming of Th1 cells, the ingestion of hyphae inhibits IL-12 and Th1 differentiation, favoring Th2 cell differentiation (Cheng et al., 2012). The Th1 and Th17 cell responses are thought to be beneficial for the host (Jacobsen et al., 2012). The different responses of dendritic cells to yeast and hyphae morphologies may thus strongly influence the clinical course of infection (Hamad, 2012; Jacobsen et al., 2012).

#### 2.2.4. Inside the phagosome

Phagocytosis is depending on the glycosylation status of the *C. albicans* cell wall, the morphology of the fungus, the hyphal length, orientation and contact of the hyphae relative to the phagocyte as well as the immune cell types and their state of activation (Whittington et al., 2014). *C. albicans* in turn has developed mechanisms to resist phagocytic killing by escaping and even killing some phagocytic cell types (Faro-Trindade and Brown, 2009; Dementhon et al., 2012; Luo et al., 2013; Vylkova and Lorenz, 2014; Wellington et al., 2014). Several phagocytes can efficiently ingest *C. albicans* yeast cells and short hyphae (Jacobsen et al., 2012; Smith and May, 2013; Whittington et al., 2014). Accordingly, a natural evasion strategy is to form long hyphae because they can not be phagocytosed for simple geometrical reasons. This is analogous to needle-shaped micro-particles which can not be engulfed either. Without intervention by the phagocytosed fungus, the phagosome matures via a series of fusion and fission events with the lysosome into the phagolysosome (Cheng et al., 2012; Brunke and Hube, 2013). However, early upon phagocytosis *C. albicans* is able to alter intracellular membrane trafficking within the phagosome by inhibiting phagosome maturation (Cheng et al., 2012; Dementhon et al., 2012; Vylkova and Lorenz, 2014). Inside the hostile environment of the phagolysosome *C. albicans* cells are killed and degraded by nutrient starvation, low pH levels, hydrolytic enzymes, antimicrobial peptides, ROS and reactive nitrogen species (NOS) (Faro-Trindade and Brown, 2009; Zipfel et al., 2011; Cheng et al., 2012; Luo et al., 2013; Mayer et al., 2013).

While neutrophils can block hyphal development (Faro-Trindade and Brown, 2009), *C. albicans* cells are able to generate hyphae within the phagolysosome of dendritic cells and macrophages allowing the fungus in some cases to kill and escape from those phagocytes (Faro-Trindade and Brown, 2009; Luo et al., 2013). Hyphal formation is depending on the pH-level (Faro-Trindade and Brown, 2009). While the acidic pH inside the phagosome should inhibit germination *C. albicans* is able to modulate the phagosomal milieu (Vylkova and Lorenz, 2014). *C. albicans* can rapidly alkalize the phagosomal environment via the arginine biosynthetic pathway (Lopez, 2013; Vylkova and Lorenz, 2014). Neutralization of the pH via the extrusion of ammonia presumably derived from the amino acid, results in the auto-induction of hyphal formation (Bain et al., 2012; Vylkova and Lorenz, 2014).

Some macrophages are able to withstand the stress of elongating *C. albicans* filaments without apparent loss of integrity (Krysan et al., 2014). In other macrophages, however, the *C. albicans* hyphal formation can provoke pyroptosis by activating the NLRP3 (NOD-like receptor family, pyrin domain containing 3) inflammasome and caspase-1. This proinflammatory, inflammasome-mediated programmed cell death pathway leads to the macrophage lysis and production of IL-1β and IL-18, allowing *C. albicans* to escape the hostile environment of the phagocyte. Early upon phagocytosis the majority of macrophage lysis is mediated by pyroptosis (Uwamahoro et al., 2014; Wellington et al., 2014). Later, a second macrophage killing phase, independent and distinct from pyroptosis, is initiated by *C. albicans* which depends on robust hyphal formation. As pyroptosis has a protective role in infections with bacterial pathogens by increasing inflammatory responses this might also be the case in *C. albicans* infections (Uwamahoro et al., 2014).

In a minority of cases phagocytosed *C. albicans* cells can escape from macrophages through non-lytic expulsion (Brunke and Hube, 2013; Lopez, 2013), also called exocytosis (Bain et al., 2012) or vomitosis (Whittington et al., 2014). This rare event is reported to occur at a low frequency but repeatedly in various experimental conditions (Bain et al., 2012). Although the underlying mechanisms are unknown (Lopez, 2013) it is observed that both, the macrophage as well as the *C. albicans* cell, remain intact and viable during phagocytosis and subsequent expulsion. This means the macrophage cell retains its phagocytic ability and is able to undergo mitosis as shown in Bain et al. (2012) while the *C. albicans* cell can perform hyphae elongation at normal rates. As non-lytic expulsion avoids lysis of the macrophage but also of the *C. albicans* cell this process may benefit both cell types (Bain et al., 2012). It is therefore not trivial to account the process as strategy to either macrophages or *C. albicans*.

#### 2.2.5. Oxidative and nitrosative stress and its detoxification

**Figure 1:**
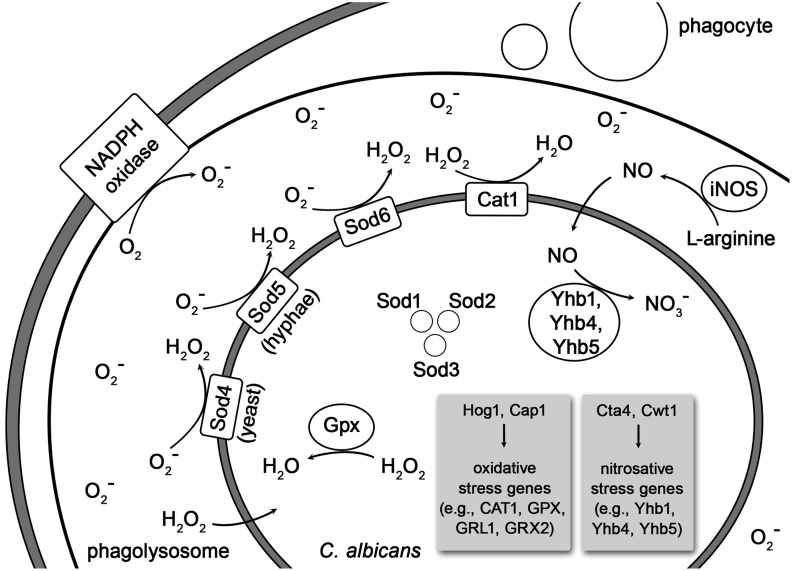
**Depiction of oxidative and nitrosative stress imposed on phagocytosed *C. albicans* and its detoxification by the fungus**. The Curved lines indicate cell membranes of the phagocyte, the phagolysosome and, most inside, the *C. albicans* cell. Abbreviations: iNOS, inducible nitric oxide synthase; Cta1, catalase 1; Sod1-6, superoxide dismutases 1–6; Gpxs, glutathione peroxidases; GRX2 and GRL1 encode glutathione reductases; Yhb1, Yhb4, and Yhb5, flavohemoglobin 1, 4, and 5; Hog1, mitogen-activated protein kinase; Cap1, adenylate cyclase-associated protein; Cta4, transcription factor; Cwt1, cell wall transcription factor.

Phagocytes can produce oxidative and nitrosative stresses to kill *C. albicans* (Wilson et al., 2009; Cheng et al., 2012; Mayer et al., 2013) (see Figure 1). The respiratory burst as summarized by Faro-Trindade and Brown (2009) is mediated via the phagocyte NADPH oxidase (Phox). This membrane-associated protein complex generates superoxide through the transfer of electrons from NADPH to O_2_. The generated superoxide (O^−^_2_) has little, if any, toxicity but can be converted to hydrogen peroxide (H_2_O_2_) and hydroxyl radicals (HO^−^) with candidacidal activity. Myeloperoxidase (MPO), an enzyme located in granules of neutrophils and in lysosomes of monocytes (and even macrophages when they scavenge it through their mannose receptors), catalyzes the further conversion of hydrogen peroxide to hypochlorous acid (HClO). The hypochlorous acid in turn is an extremely toxic and effective candidacidal oxidant. The production of nitric oxide (NO) is induced by the inducible nitric oxide synthase (iNOS or NOS2) through the oxidative deamination of L-arginine. The NO itself has poor candidacidal activity but can further react with the superoxide (O^−^_2_), generated by the respiratory burst (Vazquez-Torres et al., 1996; Faro-Trindade and Brown, 2009). The so produced peroxynitrite (ONOO^−^), an unstable structural isomer of nitrate (NO^−^_3_), is very effective at killing *C. albicans* (Vazquez-Torres et al., 1996; Faro-Trindade and Brown, 2009; Cheng et al., 2012). As the production of ROS and nitrosative stress are major antifungal mechanisms in phagocytes, *C. albicans* possesses several defense strategies to counteract the oxidative and nitrosative stresses (Cheng et al., 2012; Mayer et al., 2013).

The response of *C. albicans* to ROS is regulated by Cap1 (adenylate cyclase-associated protein 1) and the MAP (mitogen-activated protein) kinase Hog1. Both proteins regulate the catalase expression in *C. albicans* (Lopez, 2013). The fungus can produce antioxidant enzymes like the catalase Cta1 and intracellular as well as extracellular superoxide dismutases (Sods) to counteract the respiratory burst (Faro-Trindade and Brown, 2009; Frohner et al., 2009; Cheng et al., 2012; Mayer et al., 2013; Miramón et al., 2013). Of the intracellular Sods, Sod1 is required for interaction with macrophages and Sod2 is necessary to resist neutrophil attack (Miramón et al., 2013). Next to the catalase Cta1, the superoxide dismutases Sod5, Sod4 (Frohner et al., 2009) and Sod6 detoxify extracellular ROS produced by macrophages (Lopez, 2013). The expression of Sods is depending on the fungal morphology. While Sod4 is expressed by *C. albicans* yeast cells, the hyphal forms express Sod5 (Miramón et al., 2013). Neutrophils also induce the expression of Sod5 even though they inhibit the yeast-to-hyphal formation in *C. albicans* (Frohner et al., 2009; Miramón et al., 2013). Furthermore, in response to incubation with neutrophils Sods, catalase, glutathione peroxidase, glutathione reductase, glutathione S-transferase and thioredoxin are strongly induced in *C. albicans* cells (Wilson et al., 2009). The superoxide detoxification generates H_2_O_2_ which is still highly toxic but subsequently eliminated by Cat1. The glutathione peroxidases (Gpxs) also detoxify H_2_O_2_ via oxidation of the thiolgroups in two glutathione molecules, which are subsequently reduced by glutathione reductases (Grxs), encoded by GRX2 and GRL1 (Miramón et al., 2013). In addition *C. albicans* up-regulates DNA damage repair systems and heat shock proteins to counteract oxidative damage to nucleic acids and proteins (Faro-Trindade and Brown, 2009). The exposure to moderate concentrations of ROS induces the entire arginine biosynthetic pathway but no other amino acid synthetic genes in phagocytosed *C. albicans* cells (Lopez, 2013).

The nitrosative stress response of *C. albicans* is mediated by the three intracellular flavohemoglobin enzymes Yhb1, Yhb4, and Yhb5 (Lopez, 2013; Mayer et al., 2013) which convert NO to less toxic NO^−^_3_ molecules (Luo et al., 2013). The nitrosative stress response is regulated by the two transcription factors Cta4 and Cwt1. While Cta4 positively regulates the transcriptional nitrosative stress response, Cwt1 negatively regulates it (Miramón et al., 2013).

#### 2.2.6. Extracellular traps

The production of extracellular traps (ETs) is a phagocytosis independent antimicrobial mechanism observed in many effector cells including neutrophils and macrophages (Pruchniak et al., 2012; Branzk and Papayannopoulos, 2013; Hahn et al., 2013; Liu et al., 2014). These fiber-like extracellular structures are induced by many different microbes including *C. albicans* (Faro-Trindade and Brown, 2009), chemicals and cytokines (Liu et al., 2014). As being significantly associated with the microbial surface ETs are thought to act as a physical barrier that prevents invading pathogens from further progressing. The formation of ETs was therefore proposed as supplementary strategy by the host defense when phagocytosis failed to eliminate the invading pathogen (Liu et al., 2014).

Neutrophil extracellular traps (NETs) occur as specialized form of neutrophils cell death and consist of DNA scaffolds with antimicrobial proteins like histones and granule proteins including myeloperoxidase, elastase, cathelicidins, cathepsin G, calprotectins and gelatinase B (Faro-Trindade and Brown, 2009; Urban et al., 2009; Moyes and Naglik, 2011; Liu et al., 2014). Releasing these effector molecules into the extracellular space allows neutrophils to efficiently trap and kill the yeast and hyphal forms of *C. albicans* (Faro-Trindade and Brown, 2009; Liu et al., 2014). While this is beneficial for the host defense, NETs also participate in propagating some autoimmune diseases such as systemic lupus erythematosus and small vessel vasculitis (Liu et al., 2014).

*C. albicans* cells also induce the formation of METs like structures (METs-LS). These METs-LS can be released by dying as well as viable macrophages and thus show more than one type of composition. While some METs-LS consist of a DNA backbone and microbicidal proteins including histone, myeloperoxidase and lysozyme, other METs-LS did not contain histone. As histones are associated with nuclear DNA it was proposed that the DNA backbone in those METs-LS without histone originates from mitochondrial DNA. In contrast to NETs, METs-LS are not capable to efficiently kill *C. albicans* cells. Instead METs-LS rather contain the invading pathogen at the infection site, thereby preventing the systemic diffusion of *C. albicans* and providing time to recruit other effector cells like neutrophils (Liu et al., 2014).

While the formation of ETs usually depends on the generation of ROS via the activation of the NADPH oxidase, *C. albicans* induces NETs and METs-LS in an ROS independent manner (Liu et al., 2014).

## 3. Computational systems biology approaches

In many fields of biology, Computational Systems Biology approaches have turned out to be very useful (Heinrich and Schuster, 1996; Klipp et al., 2011). Various Systems Biology methods for understanding and predicting fungal virulence have been reviewed by Tierney et al. (2014). For other organisms, it has been shown that network analyses are useful to describe and understand the manifold interactions between a pathogen and its host (Naseem et al., 2012).

The basis for many Systems Biology approaches is provided by high throughput data. There are several studies regarding the omics of *Candida*. For instance, eight *Candida* genomes were compared by Butler et al. (2009). They found large families and genome expansions regarding the cell wall, secreted proteins and transporters, in particular in pathogenic species. These adaptations seemed thus to be associated with virulence.

Comprehensive transcriptome data were collected by Bruno et al. (2010). Measuring gene expression they identified 602 novel transcriptionally active regions. Conditions included hyphae-induction, tissue culture, high and low oxidative stress, nitrosative stress as well as cell wall damage-inducing conditions.

Regarding the omics of infection, there are in principle also dual sequencing approaches feasible but this is not really explored yet. Instead, Liu et al. (2015) investigated the host response to *C. albicans* infection in various niches and derived exciting results. Network analysis, siRNA knock down and RNAseq data identified new host signaling pathways under infection such as platelet-derived growth factor BB (PDGF BB) and neural precursor-cell-expressed developmentally down-regulated protein 9 (NEDD9). Both proteins regulate the uptake of *C. albicans* by host cells.

Regarding metabolite data, there is a lipidomics study by Singh et al. (2013) studying changes in *C. albicans* caused by fluconazole, a drug against candidiasis. Under this treatment, *C. albicans* shows an increased sterol content and depleted sphingolipid levels in case of azole resistance.

As evidenced by omics data many mechanisms and phenomena in biology (e.g., entangled positive and negative feedback loops) are so complex that they cannot be understood by intuition. This is one reason for the ever increasing importance of computer simulations. A first and important step is to explain known phenomena on theoretical grounds, thus helping us to understand them. The usefulness of this aspect of Computational Systems Biology should not be underestimated. A famous early example is the Michaelis-Menten kinetics. This formal approach helps us to understand the role of the association and dissociation of the enzyme-substrate complex. It has a predictive aspect because it allows one to calculate the reaction velocity even for substrate concentrations for which no measurement has been performed for a specific enzyme so far.

The most ambitious goal is to predict hitherto unknown properties, interactions and behaviors. Several studies show that Computational Systems Biology approaches can generate clear testable predictions that could later be confirmed in experiments (Schuster et al., 2006) or highlight new working hypotheses for *in vitro* experiments (Siegismund et al., 2014a,b). The latter study investigates the early colonization of bacteria on different biomaterials typically used for implants. Automated images analysis of CLSM images and point-pattern analysis were applied to show material-induced switches from bacterial adhesion to colony growth on biomaterials. By two- or three-dimensional modeling, e.g., using cellular automata or agent-based models, the adhesion of pathogens and/or epithelial cells of the host on implant surfaces can be simulated. For the case of bacteria, see Siegismund et al. (2014b). This helps us to understand the onset of disease in the case where the pathogens win this “race for the surface” (Subbiahdoss et al., 2009) and the avoidance of disease in the case where the host cells win. This will also help to devise novel therapeutic strategies, as an appropriate surface structure of the implants can diminish adhesion by pathogens. A model for the thermal adaptation of *Candida* based on a differential equation system was proposed by Leach et al. (2012). That model appropriately describes the defense-evasion pair “fever—heat shock response.” Moreover, other modeling techniques have been used to study *Candida* infections, such as Bayesian modeling (Shankar et al., 2015) and dynamic interactive infectious networks (Chen and Wu, 2014).

Several defense and evasion mechanism have been described by mathematical modeling. For example, the action of degradative enzymes can be simulated by kinetic models of metabolic networks (Heinrich and Schuster, 1996). Kinetic models of tryptophan metabolism (Stavrum et al., 2013) and of multi-drug resistance pumps have been published (Westerhoff et al., 2000). A large body of literature on the modeling of biofilm formation is available (Audretsch et al., 2013), though mostly on bacterial rather than fungal biofilms, for a review see Horn and Lackner (2014). Moreover, a gene regulatory network was inferred (Tierney et al., 2012). All of those modeling techniques could in principle be applied to investigate *C. albicans'* interactions with the host.

In the present chapter, we outline the modeling methods based on game theory and agent-based models in more detail.

### 3.1. Game theory

Metaphorically, the struggle between pathogens and the human immune system can be considered as a game in which each player attempts to win (Renaud and De Meeus, 1991; Hummert et al., 2014). This metaphor is quite useful because it allows one to understand that struggle as an extended optimization process. The extension is that the two counterparts (players) cannot always reach the optimal state because they may hinder each other in reaching it. Thus, suboptimal states can result (Hofbauer and Sigmund, 1998).

A considerable number of game-theoretical models of bacterial and viral infections have been proposed, for a review see Hummert et al. (2014), while fungal infections are the subject of such studies to a lesser extent so far. To our knowledge, Hummert et al. (2010) were the first to present a game-theoretical model of the interaction of *C. albicans* with the human immune system, in particular, with human macrophages. The simplifying assumption used was that *C. albicans* has two strategies when engulfed by macrophages: avoiding lysis transiently (silencing) or undergoing a morphological switch to form hyphae and escaping (piercing). The latter situation corresponds to the defense-evasion pair “phagocytosis—pyroptosis.” In the approach by Hummert and coworkers, different *Candida* cells are considered as players while the macrophage was considered as a constant environment. Thus, a symmetric game results and the fitness matrix can be written as follows:


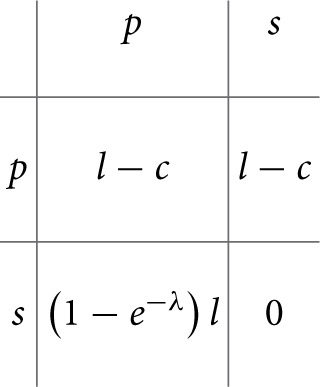


where *s* and *p* stand for the silencing and piercing strategies, respectively, *l* stands for the benefit of surviving, *c* for having payed the costs for piercing and λ denotes the average number of engulfed cells. A Poisson distribution for the number of ingested *C. albicans* yeast cells was assumed.

Every entry in the fitness matrix gives the payoff of an individual playing a row strategy against a pure population playing the column strategy. Under certain parameter conditions, a pure piercing population can exist. For other parameter values, a mixed evolutionary stable strategy (ESS) results, which corresponds to coexistence of silencing and piercing cells. The silencing cells then benefit from the efforts made by the piercing cells. In game-theoretical terms, this is a hawk-dove game (Hofbauer and Sigmund, 1998; Stark, 2010). Both of the above-mentioned outcomes are in good agreement with experimental observations, because two different karyotypes had been found (Tavanti et al., 2006).

A related model was established to describe the switch from yeast to hyphae upon invasion of human tissues (rather than inside macrophages) (Tyc et al., 2014). These authors extended the model by differential equations, allowing them to describe the dynamic behavior. Two situations are compared: cooperation between yeast and hyphae forms, meaning that the yeast form will also benefit when some cells switch to become hyphae, and competition, in which coexistence of yeast and hyphal cells pays off only to the hyphae. The model predicts that cooperation among fungal cells occurs in mild infections and an enhanced tendency to invade the host is associated with the competitive behavior (Tyc et al., 2014).

Recent progress investigated the iterative Prisoners' Dilemma. Interestingly, there is an incentive for cooperativity under these circumstances. It is worthwhile to use these mathematical insights in the context of recurrent *Candida* infections (an often happening medical condition). As known for different bacterial strains such as *Pseudomonas* in Mucoviscidosis there should be some signs for selection for mitigated strains in such repeated *Candida* infections. Furthermore, dictator strategies force a certain win or loss on the opponent, no matter which strategy is chosen (Axelrod and Hamilton, 1981). Such a way of action should be the typical strategy of the immune system in the healthy person but has not yet been extensively investigated, in particular as such healthy persons rarely undergo clinical investigation.

In contrast to the game between different *Candida* cells, the “game” between the immune system and pathogens is an asymmetric game. A pioneering paper on that type of description was published by Renaud and De Meeus (1991) for the general case of any pathogen. The two players can choose between an aggressive strategy (called the “killer” strategy) that seeks to eliminate the adversary and a less aggressive strategy (“diplomat”).

Renaud and De Meeus (1991) wrote down a rather general payoff matrix involving several parameters. To illustrate the idea, we here give a more specific matrix. However, the concrete numbers do not matter as long as they fulfill certain order relations. The entries in the matrix can be explained as follows. If both sides adopt the “killer” strategy, they win with a certain probability and have to afford the costs for that aggressive behavior. This is here quantified by 1 for either side. If both adopt the “diplomat” strategy, they can coexist and need not afford the costs for aggression. Thus, they can gain, say, 5 points each. If the host and parasite play “killer” and “diplomat,” respectively, the latter will be eliminated (payoff of 0). The host survives but has to afford some costs, so that its payoff is between 1 and 5 (here assumed to be 3). In the converse situation, the host will die (or at least become very sick), here quantified by 0. The parasite has a benefit *b* and some cost *c*.


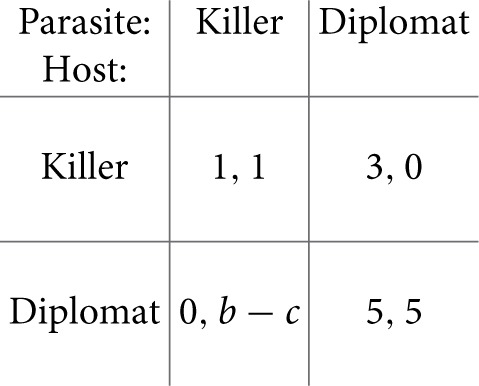


The type of game depends on the difference *b* − *c*. If it is less than 5, there are two stable Nash equilibria on the main diagonal: “killer, killer” and “diplomat, diplomat.” Loosely speaking, we can denote them by “war” and “peace.” The game is then related to the coordination game, in which the two players have to coordinate with each other to select among two symmetric Nash equilibria (Stark, 2010). Although the peaceful situation is better for both of them, they can get stuck in the war because neither side can leave it unilaterally without decreasing its payoff even more. It is worth noting that the non-lytic expulsion of *C. albicans* cells from macrophages mentioned in Section 2.2.4 can be considered as a peaceful situation as well.

If *b* − *c* > 5 (that is, high benefit or low cost of “killer” strategy for the parasite), peace is no longer stable because there is an incentive for the parasite to switch to the “killer” strategy. The state “diplomat, killer” is not, however, stable either because the host will then switch to “killer” as well. Thus, only war is stable. This change of Nash equilibria is a suitable model for the change from immunocompetent to susceptible hosts (e.g., after antibiotics treatment due to change in bacterial flora). The cost *c* for the parasite to invade the host then decreases, so that *b* − *c* can exceed the critical threshold.

The situation where only war is stable in the killer-diplomat game is quite paradoxical because both sides would be better off if they were in peace with each other. This is reminiscent of the famous Prisoners' Dilemma, which is a symmetric game (Stark, 2010). In fact, the cause for instability is similar in both games: it is the temptation (incentive) to leave the mutually beneficial state. The difference between the two games is that, in the Prisoners' Dilemma, both players are tempted in this way while in the killer-diplomat game, there is a temptation for the parasite only.

### 3.2. Agent-based modeling

Agent-based models (ABMs) have become a powerful tool for tackling complex systems, where the individuality, temporal state and spatial distribution of its players may be of importance. They are typically characterized by numerous interacting entities, often called agents or individuals (depending on the discipline so that the term individual-based model (IBM) is used as well). They pursue certain objectives (e.g., increasing fitness, yield, status) by following, more or less, simple structured rules. These agents can be mobile or stationary units within a continuous or discrete environment defined by three, two, one or even no spatial dimension. *In silico* environments without any dimension simply imply that the modeled system behavior is presumably independent of any spatial scale. Including more dimensions assumes that this may be of importance for the behavior of the system: A model investigating the hunting strategies of a terrestrial predator may be sufficiently described by a two-dimensional environment. Whereas a third dimension has to be considered simulating the movement of immune-cells through different tissues or in the blood.

The philosophy of ABMs is to slice problems on the macro-level down to simple interaction- and reaction-rules of players on a micro-level. For example, patterns occurring on the population level are transferred to properties and the behavior of single individuals. Diseases of an individual can be explained by the malfunction or disorder of organs and tissues. Often the macro-level behavior of a system cannot be foreseen by only summing up the rules of players. Instead, patterns may arise from the complex interdigitation of state-dependent behavior of its entities, an effect called emergence.

Resolving a macro-level pattern (emergence of a certain behavior) to a lower complexity level comes at the price of a detailed knowledge of the individuals properties and behavioral strategies, which have to be precisely formulated. Especially models representing a biological system frequently deal with several involved types of agents (e.g., food-webs, stability of ecosystems) and numerous interactions often require a bottom-up modeling approach with a deep knowledge of individual properties. Thus, ABMs are typically hungry for data (e.g., thresholds for reaction to signals, kinetic parameters) and computationally expensive due to, e.g., a frequent use of random number generators to induce local and individual stochasticity. Beside classical experimental approaches (e.g., for determining growth-rates), image- and video-derived data offer a valuable complementary solution to fill this gap of knowledge (Mech et al., 2011, 2014).

Deviating from an equation-based modeling approach typical ABMs show a considerable set of non-redundant rules. This often poses difficulties to communicate ABMs. Grimm and colleagues addressed this obstructive problem by proposing a standardized- and later updated protocol to formalize the descriptions of ABMs (Grimm et al., 2006, 2010).

**Figure 2:**
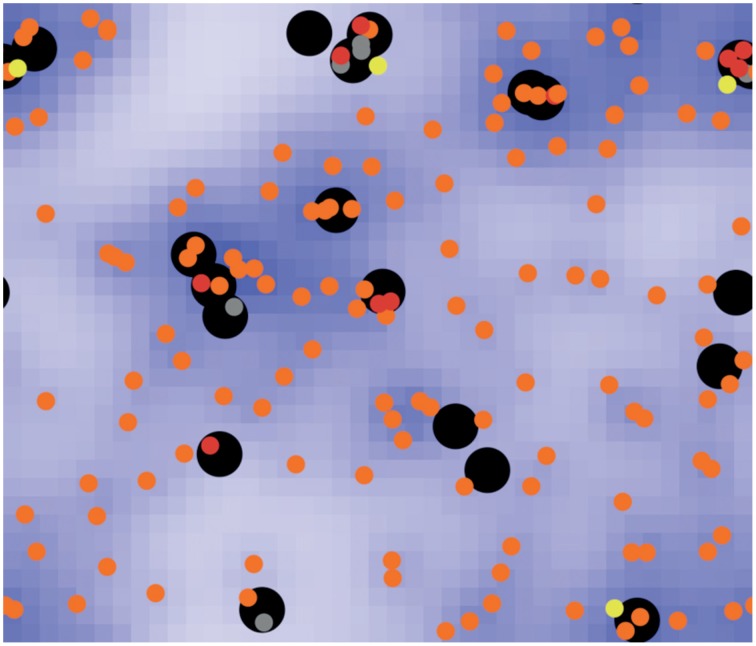
**Screenshot of a typical ABM simulation (taken from Tokarski et al., 2012), displaying the hunt of free-moving neutrophil agents (black circles) for immobile spore agents of *Aspergillus fumigatus* in human lung tissue**. Spores can be free (orange), temporarily dragged (yellow) or caught (red) by a neutrophil agent. Dragged spores may be released with a certain probability or caught and phagocytosed (gray). Neutrophil agents are able to detect chemokines (blue), released by spores during sporulation, and may adjust their movement accordingly.

Tokarski et al. (2012) investigated several hunting strategies of alveolar macrophages for fungal spores of *Aspergillus fumigatus*. The clearing efficiency of the immune system represented the emergent property of this system. Different scenarios of interactions between both players were tested, e.g., random walk of macrophages; detection and guidance of macrophages along local gradients of degradation products, indicating sporulation of spores and positive feedback activation of macrophages which already detected fungal spores. This approach exemplarily shows that biological systems above a certain degree of complexity, would be hard, if not impossible, to handle with an equation-based model. System properties may only arise at such a high complexity, e.g., by the local, state-dependent interactions of several agents (see Figure 2).

Due to its strengths in representing complex systems, ABMs show a broad field of scientific applications incorporating ecological (DeAngelis and Grimm, 2014) and microbial questions (Kreft et al., 2013).

ABM approaches helped to understand the epidemic spread of diseases, e.g., influenza (Milne et al., 2008; Laskowski et al., 2011) and Ebola (Merler et al., 2015) or, the microbial resistance to antibiotics in *Staphylococcus aureus* (Macal et al., 2014). But were also utilized to test the efficiency of counter-strategies for disease control (Borkowski et al., 2009; Tian et al., 2013; Havas et al., 2014).

Ideally ABMs can predict biological mechanisms or strategies which were unknown from wet-lab experiments, showing that both approaches are not competitive but complementary. Pollmächer and Figge (2014) investigated migration modes of macrophages and predicted that their efficiency of finding conidia could only be explained by the release of chemotactic signals from epithelial cells associated with *Aspergillus fumigatus*.

The human immune defense system consists of a dense mesh of state-dependent interactions between numerous types of players (e.g., pathogens, neutrophils or dendritic cells). Cascades of signal molecules are steering the induction and inhibition of cell responses and locally trigger the mobilization of different defense levels (e.g., passive, innate and acquired).

Folcik et al. (2007) examined the complex interplay between the innate and adaptive parts of the immune system. The focus was on the qualitative response to a viral infection. That work showed that all parts of the immune system are non-redundant and deficiency in any components increased the probability of failure to clear the simulated viral infection.

Baldazzi et al. (2006) investigated anti-HIV therapy with a immune-system model of multiple immune-cell types. ABMs typically show a high degree of specificity, thus representing one specific issue of a complex system in detail. Thus, ABMs are often less general and hard to transfer to similar questions. Examples addressing specific pathogens are: *Clostridium* (Peer and An, 2014), *Pseudomonas aeruginosa* (Seal et al., 2011), *Leishmania* (Dancik et al., 2010), *Helicobacter pylori* (Carbo et al., 2013), and *Aspergillus fumigatus* (Tokarski et al., 2012; Pollmächer and Figge, 2014). Tyc and Klipp (2011) suggested how to combine the complex behaviors of both, the host and the pathogen. Extensive reviews regarding ABMs of the immune system can be found in Chavali et al. (2008); Bauer et al. (2009); Li et al. (2008); Forrest and Beauchemin (2007).

## 4. Discussion

In this review, we have given an overview of the immune defense mechanisms of the human host against *C. albicans* and the evasion mechanisms of the fungus to escape, circumvent or counteract the immune response. Both the terms defense and evasion are here used in a wide sense and may include attack mechanisms. While earlier reviews have given an overview of experimental observations on *C. albicans* defense and evasion strategies, we present here an integrative synthesis of experimental observation and theoretical modeling of infection strategies of *C. albicans*. Our review extends previous efforts on this topic (Zipfel et al., 2013).

On the basis of the list of mechanisms and strategies, given in Tables 1, 2, it is of interest to search for even higher levels of interaction, that is, whether there are cascades including counter-counter defenses. For example *Streptomyces clavuligerus* produces both penicillin and clavulanic acid, a β-lactamases inhibitor (Reading and Cole, 1977; Knowles, 1985). Clavulanic acid and other β-lactamase inhibitors like sulbactam and tazobactam, limit the destructive action of β-lactamases from bacteria against β-lactam compounds such as penicillins and cephalosporins (Williams, 1997). Thus, there are three levels in the case of *Streptomyces clavuligerus*: penicillin as a defense chemical, β-lactamases as an evasion (counter-defense) mechanism by bacteria and clavulanic acid as a counter-counter defense. To our knowledge, no counter-counter defense is known in the case of *C. albicans* so far. However, Qiao et al. (2013) showed that other eukaryotic pathogens, i.e., oomycetes, are able to suppress RNA silencing, for a review see Pumplin and Voinnet (2013). Examples for counter-counter defense strategies are also known from plant-virus interactions, i.e., by antagonizing the virus-induced downregulation of RNA silencing by the plant (Sansregret et al., 2013). An intriguing question in the microbiology of pathogens is: How deep such an arms race of a host-pathogen interaction may evolve? Or, in other words: Are organisms rather selected for a counter-counter defense or an evolutionary novel mechanism of direct defense. The efficiency of multiple and complex layers of defense and counter-defense can be described mathematically by methods from Operations Research (Abt, 1987).

As the examples given above like macrophage phagocytosis and pyroptosis show, it can be hard to predict the outcome of the struggle between the human immune system and *C. albicans*. A special focus of our review therefore lies on the discussion of various Systems Biology approaches. Those are undoubtedly a promising tool to represent complex host-pathogen interactions and allow for the emergence of observed *in vivo* outcomes and for extensive testing scenarios (e.g., medication, drug testing, cross-effects). For example the acquisition of human complement regulators to the cell surface can be considered as a molecular mimicry. Mathematical models of mimicry in higher organisms can be adapted to describe this phenomenon. Systems Biology approaches are instrumental for questions which are hard to conduct solely in laboratory experiments. Nevertheless, theoretical approaches have to be substantially supported and completed by *in vivo* and *in vitro* approaches.

In Tables 1, 2, both specialist and generalist effector mechanisms can be seen. One example for a generalist effector is the *C. albicans* protein Pra1 as it exerts several effects. In the terminology of networks analysis, Pra1 is a hub. Accordingly, it is of interest in future studies to analyze how complex and entangled the network of interactions is, whether it is scale-free or has small-world properties etc. (Yook et al., 2004). These properties are relevant in view of robustness against errors and mutations (Albert et al., 2000).

Another interesting question is how the host protects itself from its own “attack” mechanisms such as oxidative and nitrosative stress. Obviously, the levels of these substances should not exceed upper limits. This, in turn, might give a chance for *C. albicans* in its evasion strategies. Moreover, an optimal trade-off between immunity and autoimmunity as in the case of NETs must be found which also implies upper limits on the degree of defense. In this context, the camouflage by *C. albicans* using factor H is worth mentioning.

On or within the human host *C. albicans* not only interacts with the host but also with all the probiotic microorganisms of the host's microflora. It is therefore worthwhile to further look into these interactions, e.g., *C. albicans'* theft of iron from siderophores produced by other microorganisms via its own siderophore uptake system. Kleptoparasitism can be investigated using game theoretic models, considering individuals as well as groups (Broom and Rychtář, 2011). To our knowledge this has not been done for *C. albicans* so far.

All these topics are prone to be analyzed by mathematical modeling and computer simulations. Some of the computational methods such as agent-based models allow one to describe both temporal and spatial aspects. Ideally, *in-silico* modeling makes it possible to reduce the number of experiments with animals and ethically questionable or prohibited experiments with humans. This helps to gain further insight and make medically important predictions, for instance regarding onset of fungal sepsis and novel intervention strategies in the immunocompromised patient.

## Author contributions

Conception and design of the investigation and work: all. Drafting the manuscript: SD, SG, SS. Revising it critically for important intellectual content and final approval of the version to be published: all. Agreement to be accountable for all aspects of the work in ensuring that questions related to the accuracy or integrity of any part of the work are appropriately investigated and resolved: all.

## Funding

Deutsche Forschungsgemeinschaft (DFG) CRC/Transregio 124 “Pathogenic fungi and their human host: Networks of interaction” subproject B1 (SD, TD, and SS), subproject C4 (CS), and subproject C6 (PFZ).

### Conflict of interest statement

The authors declare that the research was conducted in the absence of any commercial or financial relationships that could be construed as a potential conflict of interest.

## References

[B1] Abt C. C. (1987). Serious Games. New York, NY: University Press of America.

[B2] Albert R. Jeong H. Barabási A.-L. (2000). Error and attack tolerance of complex networks. Nature 406, 378–382. 10.1038/35019019 10935628

[B3] Audretsch C. Lopez D. Srivastava M. Wolz C. Dandekar T. (2013). A semi-quantitative model of quorum-sensing in *Staphylococcus aureus*, approved by microarray meta-analyses and tested by mutation studies. Mol. Biosyst. 9, 2665–2680. 10.1039/c3mb70117d 23959234

[B4] Axelrod R. Hamilton W. D. (1981). The evolution of cooperation. Science 211, 1390–1396. 10.1126/science.7466396 7466396

[B5] Baillie G. S. Douglas L. J. (1998). Effect of growth rate on resistance of *Candida albicans* biofilms to antifungal agents. Antimicrobial Agents Chemother. 42, 1900–1905. 9687381 10.1128/aac.42.8.1900 PMC105707

[B6] Baillie G. S. Douglas L. J. (1999). Role of dimorphism in the development of *Candida albicans* biofilms. J. Med. Microbiol. 48, 671–679. 10.1099/00222615-48-7-671 10403418

[B7] Bain J. M. Lewis L. E. Okai B. Quinn J. Gow N. A. Erwig L.-P. (2012). Non-lytic expulsion / exocytosis of *Candida albicans* from macrophages. Fungal Genet. Biol. 49, 677–678. 10.1016/j.fgb.2012.01.008 22326419 PMC3430864

[B8] Baldazzi V. Castiglione F. Bernaschi M. (2006). An enhanced agent based model of the immune system response. Cell. Immunol. 244, 77–79. 10.1016/j.cellimm.2006.12.006 17416357

[B9] Bauer A. L. Beauchemin C. A. A. Perelson A. S. (2009). Agent-based modeling of host-pathogen systems: the successes and challenges. Inf. Sci. 179, 1379–1389. 10.1016/j.ins.2008.11.012 20161146 PMC2731970

[B10] Borkowski M. Podaima B. W. McLeod R. D. (2009). Epidemic modeling with discrete-space scheduled walkers: extensions and research opportunities. BMC Public Health 9(Suppl. 1):S14. 10.1186/1471-2458-9-S1-S14 19922684 PMC2779502

[B11] Branzk N. Papayannopoulos V. (2013). Molecular mechanisms regulating NETosis in infection and disease. Semin. Immunopathol. 35, 513–530. 10.1007/s00281-013-0384-6 23732507 PMC3685711

[B12] Broom M. Rychtář J. (2011). Kleptoparasitic melees-modelling food stealing featuring contests with multiple individuals. Bull. Math. Biol. 73, 683–699. 10.1007/s11538-010-9546-z 20467823

[B13] Brunke S. Hube B. (2013). Two unlike cousins: *Candida albicans* and *C. glabrata* infection strategies. Cell. Microbiol. 15, 701–708. 10.1111/cmi.12091 23253282 PMC3654559

[B14] Bruno V. M. Wang Z. Marjani S. L. Euskirchen G. M. Martin J. Sherlock G.. (2010). Comprehensive annotation of the transcriptome of the human fungal pathogen *Candida albicans* using RNA-seq. Genome Res. 20, 1451–1458. 10.1101/gr.109553.110 20810668 PMC2945194

[B15] Butler G. Rasmussen M. D. Lin M. F. Santos M. A. Sakthikumar S. Munro C. A.. (2009). Evolution of pathogenicity and sexual reproduction in eight *Candida* genomes. Nature 459, 657–662. 10.1038/nature08064 19465905 PMC2834264

[B16] Carbo A. Bassaganya-Riera J. Pedragosa M. Viladomiu M. Marathe M. Eubank S. (2013). Predictive computational modeling of the mucosal immune responses during *Helicobacter pylori* infection. PLoS ONE 8:e73365 10.1371/journal.pone.0073365 24039925 PMC3764126

[B17] Chandra J. Kuhn D. M. Mukherjee P. K. Hoyer L. L. McCormick T. Ghannoum M. A. (2001). Biofilm formation by the fungal pathogen *Candida albicans*: development, architecture, and drug resistance. J. Bacteriol. 183, 5385–5394. 10.1128/JB.183.18.5385-5394.2001 11514524 PMC95423

[B18] Chavali A. K. Gianchandani E. P. Tung K. S. Lawrence M. B. Peirce S. M. Papin J. A. (2008). Characterizing emergent properties of immunological systems with multi-cellular rule-based computational modeling. Trends Immunol. 29, 589–599. 10.1016/j.it.2008.08.006 18964301

[B19] Chen B.-S. Wu C.-C. (2014). A systems biology approach to study systemic inflammation, in Immunoinformatics (Methods in Molecular Biology), eds De R. K. Tomar N. (New York, NY: Springer), 403–416. 10.1007/978-1-4939-1115-8_23 25048138

[B20] Cheng S.-C. Joosten L. A. Kullberg B.-J. Netea M. G. (2012). Interplay between *Candida albicans* and the mammalian innate host defense. Infect. Immun. 80, 1304–1313. 10.1128/IAI.06146-11 22252867 PMC3318407

[B21] Cheng S.-C. Van de Veerdonk F. Smeekens S. Joosten L. A. Van der Meer J. W. Kullberg B.-J.. (2010). *Candida albicans* dampens host defense by downregulating IL-17 production. J. Immunol. 185, 2450–2457. 10.4049/jimmunol.1000756 20624941

[B22] Cheng S.-C. van de Veerdonk F. L. Lenardon M. Stoffels M. Plantinga T. Smeekens S.. (2011). The dectin-1/inflammasome pathway is responsible for the induction of protective T-helper 17 responses that discriminate between yeasts and hyphae of *Candida albicans*. J. Leukoc. Biol. 90, 357–366. 10.1189/jlb.1210702 21531876 PMC3513931

[B23] Chin V. K. Foong K. J. Maha A. Rusliza B. Norhafizah M. Chong P. P. (2014). Early expression of local cytokines during systemic *Candida albicans* infection in a murine intravenous challenge model. Biomed. Rep. 2, 869–874. 10.3892/br.2014.365 25279161 PMC4179772

[B24] Collette J. R. Lorenz M. C. (2011). Mechanisms of immune evasion in fungal pathogens. Curr. Opin. Microbiol. 14, 668–675. 10.1016/j.mib.2011.09.007 21955887

[B25] Curtis M. M. Way S. S. (2009). Interleukin-17 in host defence against bacterial, mycobacterial and fungal pathogens. Immunology 126, 177–185. 10.1111/j.1365-2567.2008.03017.x 19125888 PMC2632692

[B26] Dancik G. M. Jones D. E. Dorman K. S. (2010). Parameter estimation and sensitivity analysis in an agent-based model of *Leishmania major* infection. J. Theor. Biol. 262, 398–412. 10.1016/j.jtbi.2009.10.007 19837088 PMC2789658

[B27] De Figueiredo L. F. Schuster S. Kaleta C. Fell D. A. (2008). Can sugars be produced from fatty acids? A test case for pathway analysis tools. Bioinformatics 24, 2615–2621. 10.1093/bioinformatics/btn500 18806269

[B28] De Lima T. M. Sampaio S. C. Petroni R. Brigatte P. Velasco I. T. Soriano F. G. (2014). Phagocytic activity of LPS tolerant macrophages. Mol. Immunol. 60, 8–13. 10.1016/j.molimm.2014.03.010 24732064

[B29] De Luca A. Zelante T. D'angelo C. Zagarella S. Fallarino F. Spreca A.. (2010). IL-22 defines a novel immune pathway of antifungal resistance. Mucosal Immunol. 3, 361–373. 10.1038/mi.2010.22 20445503

[B30] DeAngelis D. L. Grimm V. (2014). Individual-based models in ecology after four decades. F1000Prime Rep. 6:39. 10.12703/P6-39 24991416 PMC4047944

[B31] Dementhon K. El-Kirat-Chatel S. Noël T. (2012). Development of an *in vitro* model for the multi-parametric quantification of the cellular interactions between *Candida* yeasts and phagocytes. PLoS ONE 7:e32621. 10.1371/journal.pone.0032621 22479332 PMC3316538

[B32] Den Hertog A. Van Marle J. Van Veen H. Van't Hof W. Bolscher J. G. Veerman E. C.. (2005). Candidacidal effects of two antimicrobial peptides: histatin 5 causes small membrane defects, but LL-37 causes massive disruption of the cell membrane. Biochem. J. 388, 689–695. 10.1042/BJ20042099 15707390 PMC1138977

[B33] d'Enfert C. (2009). Hidden killers: persistence of opportunistic fungal pathogens in the human host. Curr. Opin. Microbiol. 12, 358–364. 10.1016/j.mib.2009.05.008 19541532

[B34] d'Ostiani C. F. Del Sero G. Bacci A. Montagnoli C. Spreca A. Mencacci A.. (2000). Dendritic cells discriminate between yeasts and hyphae of the fungus *Candida albicans* implications for initiation of T helper cell immunity *in vitro* and *in vivo*. J. Exp. Med. 191, 1661–1674. 10.1084/jem.191.10.1661 10811860 PMC2193147

[B35] Eyerich S. Wagener J. Wenzel V. Scarponi C. Pennino D. Albanesi C.. (2011). IL-22 and TNF-α represent a key cytokine combination for epidermal integrity during infection with *Candida albicans*. Eur. J. Immunol. 41, 1894–1901. 10.1002/eji.201041197 21469124

[B36] Faro-Trindade I. Brown G. D. (2009). Interaction of *Candida albicans* with phagocytes, in Phagocyte-Pathogen Interactions: Macrophages and the Host Response to Infection, eds Russell D. G. Gordon S. (Washington, DC: ASM Press), 437–451.

[B37] Filler S. G. (2013). Can host receptors for fungi be targeted for treatment of fungal infections? Trends Microbiol. 21, 389–396. 10.1016/j.tim.2013.05.006 23796589 PMC3735786

[B38] Finkel J. S. Mitchell A. P. (2011). Genetic control of *Candida albicans* biofilm development. Nat. Rev. Microbiol. 9, 109–118. 10.1038/nrmicro2475 21189476 PMC3891587

[B39] Folcik V. A. An G. C. Orosz C. G. (2007). The basic immune simulator: an agent-based model to study the interactions between innate and adaptive immunity. Theor. Biol. Med. Model. 4:39. 10.1186/1742-4682-4-39 17900357 PMC2186321

[B40] Forrest S. Beauchemin C. (2007). Computer immunology. Immunol. Rev. 216, 176–197. 10.1111/j.1600-065X.2007.00499.x 17367343

[B41] Frohner I. E. Bourgeois C. Yatsyk K. Majer O. Kuchler K. (2009). *Candida albicans* cell surface superoxide dismutases degrade host-derived reactive oxygen species to escape innate immune surveillance. Mol. Microbiol. 71, 240–252. 10.1111/j.1365-2958.2008.06528.x 19019164 PMC2713856

[B42] Ganguly S. Mitchell A. P. (2011). Mucosal biofilms of *Candida albicans*. Curr. Opin. Microbiol. 14, 380–385. 10.1016/j.mib.2011.06.001 21741878 PMC3159763

[B43] Gow N. A. van de Veerdonk F. L. Brown A. J. Netea M. G. (2012). *Candida albicans* morphogenesis and host defence: discriminating invasion from colonization. Nat. Rev. Microbiol. 10, 112–122. 10.1038/nrmicro2711 22158429 PMC3624162

[B44] Grimm V. Berger U. Bastiansen F. Eliassen S. Ginot V. Giske J. (2006). A standard protocol for describing individual-based and agent-based models. Ecol. Model. 198, 115–126. 10.1016/j.ecolmodel.2006.04.023

[B45] Grimm V. Berger U. DeAngelis D. L. Polhill J. G. Giske J. Railsback S. F. (2010). The ODD protocol: a review and first update. Ecol. Model. 221, 2760–2768. 10.1016/j.ecolmodel.2010.08.019

[B46] Gropp K. Schild L. Schindler S. Hube B. Zipfel P. F. Skerka C. (2009). The yeast *Candida albicans* evades human complement attack by secretion of aspartic proteases. Mol. Immunol. 47, 465–475. 10.1016/j.molimm.2009.08.019 19880183

[B47] Hahn S. Giaglis S. Chowdury C. S. Hösli I. Hasler P. (2013). Modulation of neutrophil NETosis: interplay between infectious agents and underlying host physiology. Semin. Immunopathol. 35, 439–453. 10.1007/s00281-013-0380-x 23649713 PMC3685704

[B48] Hamad M. (2012). Innate and adaptive antifungal immune responses: partners on an equal footing. Mycoses 55, 205–217. 10.1111/j.1439-0507.2011.02078.x 21815944

[B49] Havas K. A. Boone R. B. Hill A. E. Salman M. D. (2014). A Brucellosis disease control strategy for the Kakheti region of the Country of Georgia: an agent-based model. Zoonoses Public Health 61, 260–270. 10.1111/zph.12066 23879523

[B50] Heinrich R. Schuster S. C. (1996). The Regulation of Cellular Systems. New York, NY: Chapman and Hall.

[B51] Hofbauer J. Sigmund K. (1998). Evolutionary Games and Population Dynamics. Cambridge: Cambridge University Press.

[B52] Horn H. Lackner S. (2014). Modeling of biofilm systems: a review, in Productive Biofilms, eds Muffler K. Ulber R. (Berlin; Heidelberg: Springer-Verlag), 53–76. 10.1007/10_2014_275 25163572

[B53] Hummert S. Bohl K. Basanta D. Deutsch A. Werner S. Theißen G.. (2014). Evolutionary game theory: cells as players. Mol. Biosyst. 10, 3044–3065. 10.1039/C3MB70602H 25270362

[B54] Hummert S. Hummert C. Schröter A. Hube B. Schuster S. (2010). Game theoretical modelling of survival strategies of *Candida albicans* inside macrophages. J. Theor. Biol. 264, 312–318. 10.1016/j.jtbi.2010.01.022 20100495

[B55] Jacobsen I. D. Wilson D. Wächtler B. Brunke S. Naglik J. R. Hube B. (2012). *Candida albicans* dimorphism as a therapeutic target. Expert Rev. Anti. Infect. Ther. 10, 85–93. 10.1586/eri.11.152 22149617

[B56] Jiménez-López C. Lorenz M. C. (2013). Fungal immune evasion in a model host-pathogen interaction: *Candida albicans* versus macrophages. PLoS Pathog. 9:e1003741. 10.1371/journal.ppat.1003741 24278014 PMC3836912

[B57] Kagami S. Rizzo H. L. Kurtz S. E. Miller L. S. Blauvelt A. (2010). IL-23 and IL-17a, but not IL-12 and IL-22, are required for optimal skin host defense against *Candida albicans*. J. Immunol. 185, 5453–5462. 10.4049/jimmunol.1001153 20921529 PMC3076054

[B58] Klipp E. Liebermeister W. Wierling C. Kowald A. Lehrach H. Herwig R. (2011). Systems Biology: A Textbook. Weinheim: Wiley VCH.

[B59] Knowles J. R. (1985). Penicillin resistance: the chemistry of β-lactamase inhibition. Acc. Chem. Res. 18, 97–104. 10.1021/ar00112a001

[B60] Korn T. Bettelli E. Oukka M. Kuchroo V. K. (2009). Il-17 and Th17 Cells. Annu. Rev. Immunol. 27, 485–517. 10.1146/annurev.immunol.021908.132710 19132915

[B61] Kreft J.-U. Plugge C. M. Grimm V. Prats C. Leveau J. H. J. Banitz T.. (2013). Mighty small: observing and modeling individual microbes becomes big science. Proc. Natl. Acad. Sci. U.S.A. 110, 18027–18028. 10.1073/pnas.1317472110 24194530 PMC3831448

[B62] Krysan D. J. Sutterwala F. S. Wellington M. (2014). Catching fire: *Candida albicans*, macrophages, and pyroptosis. PLoS Pathog. 10:e1004139. 10.1371/journal.ppat.1004139 24967821 PMC4072798

[B63] Kumar V. Sharma A. (2010). Neutrophils: cinderella of innate immune system. Int. Immunopharmacol. 10, 1325–1334. 10.1016/j.intimp.2010.08.012 20828640

[B64] Kwak M.-K. Ku M. Kang S.-O. (2014). NAD^+^-linked alcohol dehydrogenase 1 regulates methylglyoxal concentration in *Candida albicans*. FEBS Lett., 588, 1144–1153. 10.1016/j.febslet.2014.02.042 24607541

[B65] Laskowski M. Demianyk B. C. P. Witt J. Mukhi S. N. Friesen M. R. McLeod R. D. (2011). Agent-based modeling of the spread of influenza-like illness in an emergency department: a simulation study. IEEE Trans. Inf. Technol. Biomed. 15, 877–889. 10.1109/TITB.2011.2163414 21813364

[B66] Leach M. D. Tyc K. M. Brown A. J. P. Klipp E. (2012). Modelling the regulation of thermal adaptation in *Candida albicans*, a major fungal pathogen of humans. PLoS ONE 7:e32467. 10.1371/journal.pone.0032467 22448221 PMC3308945

[B67] Li R. Kumar R. Tati S. Puri S. Edgerton M. (2013). *Candida albicans* flu1-mediated efflux of salivary histatin 5 reduces its cytosolic concentration and fungicidal activity. Antimicrobial Agents Chemother. 57, 1832–1839. 10.1128/AAC.02295-12 23380720 PMC3623299

[B68] Li Y. Nguyen M. H. Cheng S. Schmidt S. Zhong L. Derendorf H.. (2008). A pharmacokinetic / pharmacodynamic mathematical model accurately describes the activity of voriconazole against *Candida* spp. *in vitro*. Int. J. Antimicrob. Agents 31, 369–374. 10.1016/j.ijantimicag.2007.11.015 18215509 PMC2367122

[B69] Liu P. Wu X. Liao C. Liu X. Du J. Shi H.. (2014). *Escherichia coli* and *Candida albicans* induced macrophage extracellular trap-like structures with limited microbicidal activity. PloS ONE 9:e90042. 10.1371/journal.pone.0090042 24587206 PMC3934966

[B70] Liu Y. Shetty A. C. Schwartz J. A. Bradford L. L. Xu W. Phan Q. T.. (2015). New signaling pathways govern the host response to *C. albicans* infection in various niches. Genome Res. 25, 679–689. 10.1101/gr.187427.114 25858952 PMC4417116

[B71] Lopez C. M. (2013). The Roles of Candida albicans Gpm1p and Tef1p in Immune Evasion and Tissue Invasion of the Human Host. Ph.D. thesis, Jena, Friedrich-Schiller-Universität Jena.

[B72] Lu Y. Su C. Unoje O. Liu H. (2014). Quorum sensing controls hyphal initiation in *Candida albicans* through Ubr1-mediated protein degradation. Proc. Natl. Acad. Sci. U.S.A. 111, 1975–1980. 10.1073/pnas.1318690111 24449897 PMC3918812

[B73] Luo S. Skerka C. Kurzai O. Zipfel P. F. (2013). Complement and innate immune evasion strategies of the human pathogenic fungus *Candida albicans*. Mol. Immunol. 56, 161–169. 10.1016/j.molimm.2013.05.218 23809232

[B74] Macal C. M. North M. J. Collier N. Dukic V. M. Wegener D. T. David M. Z.. (2014). Modeling the transmission of community-associated methicillin-resistant *Staphylococcus aureus*: a dynamic agent-based simulation. J. Transl. Med. 12:124. 10.1186/1479-5876-12-124 24886400 PMC4049803

[B75] Martin H. L. Richardson B. A. Nyange P. M. Lavreys L. Hillier S. L. Chohan B.. (1999). Vaginal Lactobacilli, microbial flora, and risk of human immunodeficiency virus type 1 and sexually transmitted disease acquisition. J. Infect. Dis. 180, 1863–1868. 10.1086/315127 10558942

[B76] Mayer F. L. Wilson D. Hube B. (2013). *Candida albicans* pathogenicity mechanisms. Virulence 4, 119–128. 10.4161/viru.22913 23302789 PMC3654610

[B77] Mech F. Thywißen A. Guthke R. Brakhage A. A. Figge M. T. (2011). Automated image analysis of the host-pathogen interaction between phagocytes and *Aspergillus fumigatus*. PLoS ONE 6:e19591. 10.1371/journal.pone.0019591 21573171 PMC3088683

[B78] Mech F. Wilson D. Lehnert T. Hube B. Figge M. T. (2014). Epithelial invasion outcompetes hypha development during *Candida albicans* infection as revealed by an image-based systems biology approach. Cytometry A 85A, 126–139. 10.1002/cyto.a.22418 24259441

[B79] Merler S. Ajelli M. Fumanelli L. Gomes M. F. C. Piontti A. P. Y. Rossi L.. (2015). Spatiotemporal spread of the 2014 outbreak of Ebola virus disease in Liberia and the effectiveness of non-pharmaceutical interventions: a computational modelling analysis. Lancet Infect. Dis. 15, 204–211. 10.1016/S1473-3099(14)71074-6 25575618 PMC4409131

[B80] Milne G. J. Kelso J. K. Kelly H. A. Huband S. T. McVernon J. (2008). A small community model for the transmission of infectious diseases: comparison of school closure as an intervention in individual-based models of an influenza pandemic. PLoS ONE 3:e4005. 10.1371/journal.pone.0004005 19104659 PMC2602849

[B81] Miramón P. Kasper L. Hube B. (2013). Thriving within the host: *Candida* spp. interactions with phagocytic cells. Med. Microbiol. Immunol. 202, 183–195. 10.1007/s00430-013-0288-z 23354731

[B82] Moyes D. L. Naglik J. R. (2011). Mucosal immunity and *Candida albicans* infection. Clin. Dev. Immunol. 2011:346307. 10.1155/2011/346307 21776285 PMC3137974

[B83] Naseem M. Philippi N. Hussain A. Wangorsch G. Ahmed N. Dandekar T. (2012). Integrated systems view on networking by hormones in *Arabidopsis* immunity reveals multiple crosstalk for cytokinin. Plant Cell 24, 1793–1814. 10.1105/tpc.112.098335 22643121 PMC3442570

[B84] Netea M. G. Brown G. D. Kullberg B. J. Gow N. A. (2008). An integrated model of the recognition of *Candida albicans* by the innate immune system. Nat. Rev. Microbiol. 6, 67–78. 10.1038/nrmicro1815 18079743

[B85] Netea M. G. Maródi L. (2010). Innate immune mechanisms for recognition and uptake of *Candida* species. Trends Immunol. 31, 346–353. 10.1016/j.it.2010.06.007 20705510

[B86] Nobile C. J. Mitchell A. P. (2006). Genetics and genomics of *Candida albicans* biofilm formation. Cell. Microbiol. 8, 1382–1391. 10.1111/j.1462-5822.2006.00761.x 16848788

[B87] Peer X. An G. (2014). Agent-based model of fecal microbial transplant effect on bile acid metabolism on suppressing *Clostridium difficile* infection: an example of agent-based modeling of intestinal bacterial infection. J. Pharmacokinet. Pharmacodyn. 41, 493–507. 10.1007/s10928-014-9381-1 25168489 PMC4210368

[B88] Pollmächer J. Figge M. T. (2014). Agent-based model of human alveoli predicts chemotactic signaling by epithelial cells during early *Aspergillus fumigatus* infection. PLoS ONE 9:e111630. 10.1371/journal.pone.0111630 25360787 PMC4216106

[B89] Pruchniak M. P. Araźna M. Demkow U. (2012). Extracellular traps formation and visualization methods. Cent. Eur. J. Immunol. 37, 81–84.

[B90] Pumplin N. Voinnet O. (2013). RNA silencing suppression by plant pathogens: defence, counter-defence and counter-counter-defence. Nat. Rev. Microbiol. 11, 745–760. 10.1038/nrmicro3120 24129510

[B91] Qiao Y. Liu L. Xiong Q. Flores C. Wong J. Shi J.. (2013). Oomycete pathogens encode RNA silencing suppressors. Nat. Genet. 45, 330–333. 10.1038/ng.2525 23377181 PMC4049077

[B92] Quintin J. Voigt J. Voort R. Jacobsen I. D. Verschueren I. Hube B.. (2014). Differential role of NK cells against *Candida albicans* infection in immunocompetent or immunocompromised mice. Eur. J. Immunol. 44, 2405–2414. 10.1002/eji.201343828 24802993

[B93] Ramage G. Martínez J. P. López-Ribot J. L. (2006). *Candida* biofilms on implanted biomaterials: a clinically significant problem. FEMS Yeast Res. 6, 979–986. 10.1111/j.1567-1364.2006.00117.x 17042747

[B94] Ramirez-Ortiz Z. G. Means T. K. (2012). The role of dendritic cells in the innate recognition of pathogenic fungi (*A. fumigatus, C. neoformans* and *C. albicans*). Virulence 3, 635–646. 10.4161/viru.22295 23076328 PMC3545945

[B95] Reading C. Cole M. (1977). Clavulanic acid: a beta-lactamase-inhibiting beta-lactam from *Streptomyces clavuligerus*. Antimicrobial Agents Chemother. 11, 852–857. 879738 10.1128/aac.11.5.852 PMC352086

[B96] Renaud F. De Meeus T. (1991). A simple model of host-parasite evolutionary relationships. parasitism: compromise or conflict? J. Theor. Biol. 152, 319–327. 1749254 10.1016/s0022-5193(05)80197-3

[B97] Sansregret R. Dufour V. Langlois M. Daayf F. Dunoyer P. Voinnet O.. (2013). Extreme resistance as a host counter-counter defense against viral suppression of RNA silencing. PLoS Pathog. 9:e1003435. 10.1371/journal.ppat.1003435 23785291 PMC3681747

[B98] Scapini P. Cassatella M. A. (2014). Social networking of human neutrophils within the immune system. Blood 124, 710–719. 10.1182/blood-2014-03-453217 24923297

[B99] Schlatter R. Philippi N. Wangorsch G. Pick R. Sawodny O. Borner C.. (2012). Integration of Boolean models exemplified on hepatocyte signal transduction. Brief. Bioinform. 13, 365–376. 10.1093/bib/bbr065 22016404

[B100] Schuster S. Klipp E. Marhl M. (2006). The predictive power of molecular network modelling - case studies of predictions with subsequent experimental verification, in Discovering Biomolecular Mechanisms with Computational Biology, ed Eisenhaber F. (Georgetown, DC: Landes Bioscience), 95–103.

[B101] Seal J. B. Alverdy J. C. Zaborina O. An G. (2011). Agent-based dynamic knowledge representation of *Pseudomonas aeruginosa* virulence activation in the stressed gut: towards characterizing host-pathogen interactions in gut-derived sepsis. Theor. Biol. Med. Model. 8:33 10.1186/1742-4682-8-33 21929759 PMC3184268

[B102] Seneviratne C. Jin L. Samaranayake L. (2008). Biofilm lifestyle of *Candida*: a mini review. Oral Dis. 14, 582–590. 10.1111/j.1601-0825.2007.01424.x 19076549

[B103] Shankar J. Solis N. V. Mounaud S. Szpakowski S. Liu H. Losada L.. (2015). Using Bayesian modelling to investigate factors governing antibiotic-induced *Candida albicans* colonization of the GI tract. Sci. Rep. 5:8131. 10.1038/srep08131 25644850 PMC4314636

[B104] Siegismund D. Schroeter A. Lüdecke C. Undisz A. Jandt K. D. Roth M.. (2014a). Discrimination between random and non-random processes in early bacterial colonization on biomaterial surfaces: application of point pattern analysis. Biofouling 30, 1023–1033. 10.1080/08927014.2014.958999 25329612

[B105] Siegismund D. Undisz A. Germerodt S. Schuster S. Rettenmayr M. (2014b). Quantification of the interaction between biomaterial surfaces and bacteria by 3-D modeling. Acta Biomater. 10, 267–275. 10.1016/j.actbio.2013.09.016 24071002

[B106] Singh A. Mahto K. K. Prasad R. (2013). Lipidomics and *in vitro* azole resistance in *Candida albicans*. OMICS 17, 84–93. 10.1089/omi.2012.0075 23374108 PMC3567621

[B107] Smith L. M. May R. C. (2013). Mechanisms of microbial escape from phagocyte killing. Biochem. Soc. Trans. 41, 475–490. 10.1042/BST20130014 23514140

[B108] Sonnenberg G. F. Fouser L. A. Artis D. (2011). Border patrol: regulation of immunity, inflammation and tissue homeostasis at barrier surfaces by IL-22. Nat. Immunol. 12, 383–390. 10.1038/ni.2025 21502992

[B109] Stark H. U. (2010). Dilemmas of partial cooperation. Evolution 64, 2458–2465. 10.1111/j.1558-5646.2010.00986.x 20199562

[B110] Stavrum A.-K. Heiland I. Schuster S. Puntervoll P. Ziegler M. (2013). Model of tryptophan metabolism, readily scalable using tissue-specific gene expression data. J. Biol. Chem. 288, 34555–34566. 10.1074/jbc.M113.474908 24129579 PMC3843069

[B111] Steele C. Fidel P. L. (2002). Cytokine and chemokine production by human oral and vaginal epithelial cells in response to *Candida albicans*. Infect. Immun. 70, 577–583. 10.1128/IAI.70.2.577-583.2002 11796585 PMC127706

[B112] Subbiahdoss G. Kuijer R. Grijpma D. W. van der Mei H. C. Busscher H. J. (2009). Microbial biofilm growth vs. tissue integration: “the race for the surface” experimentally studied. Acta Biomater. 5, 1399–1404. 10.1016/j.actbio.2008.12.011 19158003

[B113] Swidergall M. Ernst A. M. Ernst J. F. (2013). *Candida albicans* mucin Msb2 is a broad-range protectant against antimicrobial peptides. Antimicrob. Agents Chemother. 57, 3917–3922. 10.1128/AAC.00862-13 23733470 PMC3719696

[B114] Szafranski-Schneider E. Swidergall M. Cottier F. Tielker D. Román E. Pla J. Ernst J. F. (2012). Msb2 shedding protects *Candida albicans* against antimicrobial peptides. PLoS Pathog. 8:e1002501. 10.1371/journal.ppat.1002501 22319443 PMC3271078

[B115] Tavanti A. Campa D. Bertozzi A. Pardini G. Naglik J. R. Barale R.. (2006). *Candida albicans* isolates with different genomic backgrounds display a differential response to macrophage infection. Microbes Infect. 8, 791–800. 10.1016/j.micinf.2005.09.016 16473540

[B116] Tian Y. Osgood N. D. Al-Azem A. Hoeppner V. H. (2013). Evaluating the effectiveness of contact tracing on tuberculosis Outcomes in Saskatchewan using individual-based modeling. Health Educ. Behav. 40, 98S–110S. 10.1177/1090198113493910 24084405

[B117] Tierney L. Linde J. Müller S. Brunke S. Molina J. C. Hube B.. (2012). An interspecies regulatory network inferred from simultaneous RNA-seq of *Candida albicans* invading innate immune cells. Front. Microbiol. 3:85. 10.3389/fmicb.2012.00085 22416242 PMC3299011

[B118] Tierney L. Tyc K. Klipp E. Kuchler K. (2014). Systems biology approaches to understanding and predicting fungal virulence, in Human Fungal Pathogens, Number XII in The Mycotaed, 2nd Edn., ed Kurzai O. (Berlin; Heidelberg: Springer-Verlag), 45–74.

[B119] Tokarski C. Hummert S. Mech F. Figge M. T. Germerodt S. Schroeter A.. (2012). Agent-based modeling approach of immune defense against spores of opportunistic human pathogenic fungi. Front. Microbiol. 3:129. 10.3389/fmicb.2012.00129 22557995 PMC3337507

[B120] Tyc K. M. Klipp E. (2011). Modeling dissemination of pathogenic fungi within a host: a cartoon for the interactions of two complex systems. J. Comput. Sci. Syst. Biol. S1:001 10.4172/jcsb.S1-001

[B121] Tyc K. M. Kühn C. Wilson D. Klipp E. (2014). Assessing the advantage of morphological changes in *Candida albicans*: a game theoretical study. Front. Microbiol. 5:41. 10.3389/fmicb.2014.00041 24567730 PMC3915147

[B122] Urban C. F. Ermert D. Schmid M. Abu-Abed U. Goosmann C. Nacken W.. (2009). Neutrophil extracellular traps contain calprotectin, a cytosolic protein complex involved in host defense against *Candida albicans*. PLoS Pathog. 5:639. 10.1371/journal.ppat.1000639 19876394 PMC2763347

[B123] Uwamahoro N. Verma-Gaur J. Shen H.-H. Qu Y. Lewis R. Lu J.. (2014). The pathogen *Candida albicans* hijacks pyroptosis for escape from macrophages. MBio 5, e00003–000014. 10.1128/mBio.00003-14 24667705 PMC3977349

[B124] Uyttenhove C. Pilotte L. Théate I. Stroobant V. Colau D. Parmentier N.. (2003). Evidence for a tumoral immune resistance mechanism based on tryptophan degradation by indoleamine 2,3-dioxygenase. Nat. Med. 9, 1269–1274. 10.1038/nm934 14502282

[B125] van de Veerdonk F. Joosten L. Netea M. (2015). The interplay between inflammasome activation and antifungal host defense. Immunol. Rev. 265, 172–180. 10.1111/imr.12280 25879292

[B126] Vazquez-Torres A. Jones-Carson J. Balish E. (1996). Peroxynitrite contributes to the candidacidal activity of nitric oxide-producing macrophages. Infect. Immun. 64, 3127–3133. 8757843 10.1128/iai.64.8.3127-3133.1996 PMC174197

[B127] Vialas V. Sun Z. Loureiro y Penha C. V. Carrascal M. Abián J. Monteoliva L.. (2014). A *Candida albicans* peptideatlas. J. Proteomics 97, 62–68. 10.1016/j.jprot.2013.06.020 23811049 PMC3951211

[B128] Voigt J. (2013). Die Rolle von NK-Zellen in der Immunantwort Gegen Candida albicans. Ph.D. thesis, Jena, Friedrich-Schiller-Universität Jena.

[B129] Vylkova S. Lorenz M. C. (2014). Modulation of phagosomal pH by *Candida albicans* promotes hyphal morphogenesis and requires Stp2p, a regulator of amino acid transport. PLoS Pathog. 10:e1003995. 10.1371/journal.ppat.1003995 24626429 PMC3953444

[B130] Wellington M. Koselny K. Sutterwala F. S. Krysan D. J. (2014). *Candida albicans* triggers NLRP3-mediated pyroptosis in macrophages. Eukaryot. Cell 13, 329–340. 10.1128/EC.00336-13 24376002 PMC3910967

[B131] Westerhoff H. Riethorst A. Jongsma A. (2000). Relating multidrug resistance phenotypes to the kinetic properties of their drug-efflux pumps. Eur. J. Biochem. 267, 5355–5368. 10.1046/j.1432-1327.2000.01559.x 10951193

[B132] Whittington A. Gow N. A. R. Hube B. (2014). From commensal to pathogen: *Candida albicans*, in Human Fungal Pathogens number XII in The Mycota, 2nd Edn., ed Kurzai O. (Berlin; Heidelberg: Springer-Verlag), 3–18.

[B133] Williams J. D. (1997). β-Lactamase inhibition and *in vitro* activity of sulbactam and sulbactam / cefoperazone. Clin. Infect. Dis. 24, 494–497. 9114205 10.1093/clinids/24.3.494

[B134] Wilson D. Thewes S. Zakikhany K. Fradin C. Albrecht A. Almeida R.. (2009). Identifying infection-associated genes of *Candida albicans* in the postgenomic era. FEMS Yeast Res. 9, 688–700. 10.1111/j.1567-1364.2009.00524.x 19473261

[B135] Yan L. Yang C. Tang J. (2013). Disruption of the intestinal mucosal barrier in *Candida albicans* infections. Microbiol. Res. 168, 389–395. 23545353 10.1016/j.micres.2013.02.008

[B136] Yook S.-H. Oltvai Z. N. Barabási A.-L. (2004). Functional and topological characterization of protein interaction networks. Proteomics 4, 928–942. 10.1002/pmic.200300636 15048975

[B137] Zelante T. Iannitti R. De Luca A. Romani L. (2011). IL-22 in antifungal immunity. Eur. J. Immunol. 41, 270–275. 10.1002/eji.201041246 21267995

[B138] Zenewicz L. A. Flavell R. A. (2011). Recent advances in IL-22 biology. Int. Immunol. 23, 159–163. 10.1093/intimm/dxr001 21393631

[B139] Zipfel P. F. Hallström T. Riesbeck K. (2013). Human complement control and complement evasion by pathogenic microbes–tipping the balance. Mol. Immunol. 56, 152–160. 10.1016/j.molimm.2013.05.222 23810413

[B140] Zipfel P. F. Skerka C. Kupka D. Luo S. (2011). Immune escape of the human facultative pathogenic yeast *Candida albicans*: the many faces of the *Candida* Pra1 protein. Int. J. Med. Microbiol. 301, 423–430. 10.1016/j.ijmm.2011.04.010 21565550

